# Supplementary material for: CRISPR activation screen identifies BCL-2 proteins and B3GNT2 as drivers of cancer resistance to T cell-mediated cytotoxicity
Source: Nat Commun. 2022 Mar 25;13:1606. doi: 10.1038/s41467-022-29205-8 (PMC8956604; doi:10.1038/s41467-022-29205-8)
Supplement: Supplementary file 1 — Supplementary Information [file 41467_2022_29205_MOESM1_ESM.pdf]

Supplementary Information

CRISPR activation screen identifies BCL-2 proteins and B3GNT2 as drivers of cancer resistance to T cell-mediated cytotoxicity

Julia Joung[1,2,3,4,5,8,†], Paul C. Kirchgatterer[1,2,3,4,5], Ankita Singh[1,2,3,4,5], Jang H. Cho[1,2,3,4,5], Suchita P. Nety[1,2,3,4,5], Rebecca C. Larson[6,7], Rhiannon K. Macrae[1,2,3,4,5], Rebecca Deasy[2], Yuen-Yi Tseng[2], Marcela V. Maus[6,7], and Feng Zhang[1,2,3,4,5,†]

[1] Department of Biological Engineering, MIT, Cambridge, MA 02139, USA

[2] Broad Institute of MIT and Harvard, Cambridge, MA 02142, USA

[3] Department of Brain and Cognitive Science, MIT, Cambridge, MA 02139, USA

[4] McGovern Institute for Brain Research at MIT, Cambridge, MA 02139, USA

[5] Howard Hughes Medical Institute, MIT, Cambridge, MA 02139, USA

[6] Department of Medicine, Massachusetts General Hospital and Harvard Medical School, Boston, MA, USA

[7] Cellular Immunotherapy Program, Cancer Center, Massachusetts General Hospital and Harvard Medical School, Boston, MA, USA

[8] Present address: Whitehead Institute, Cambridge, MA 02142, USA

[†] Correspondence should be addressed to julia@joung.science (J.J.) and zhang@broadinstitute.org (F.Z.).

Supplementary Figures

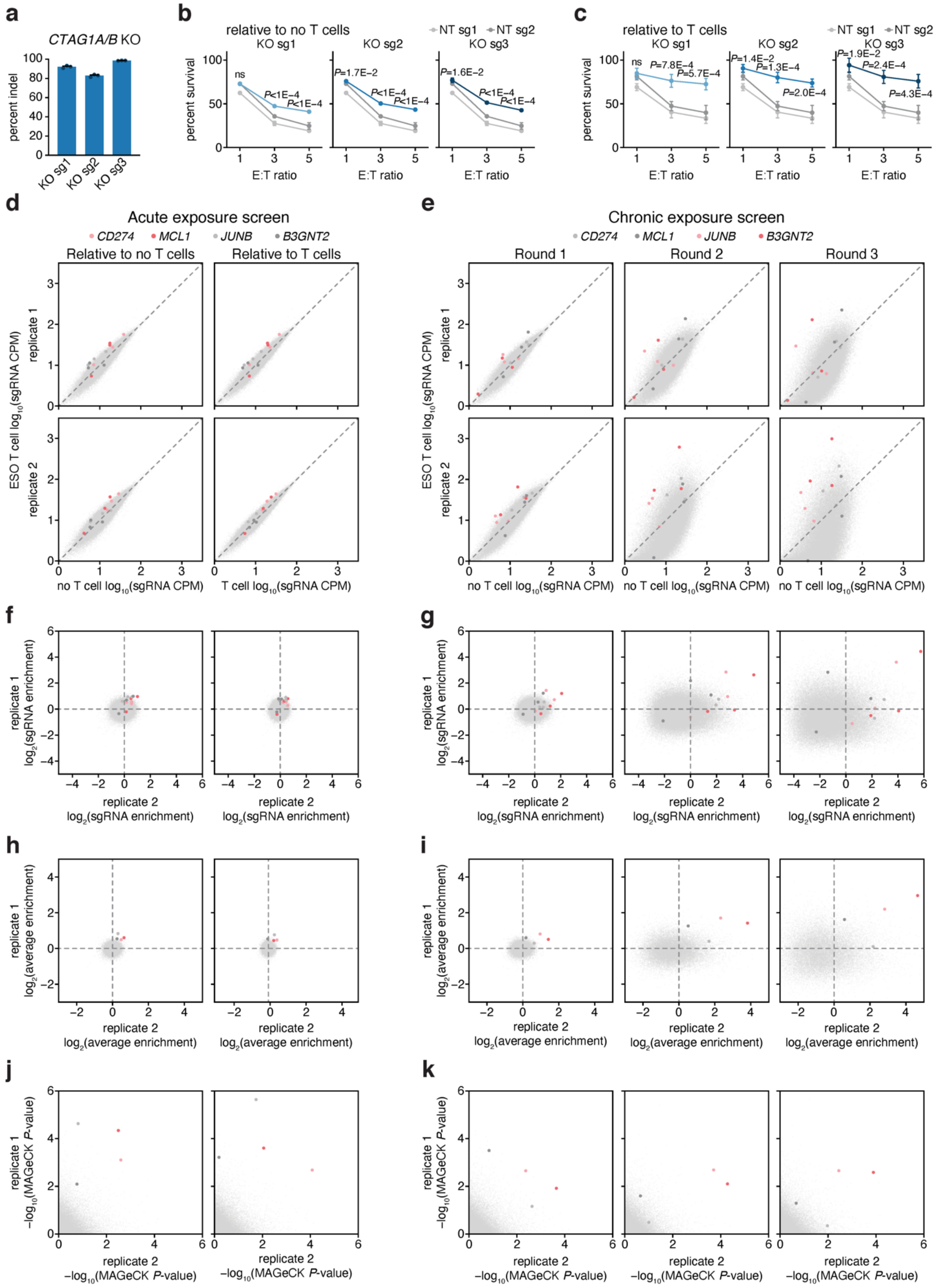

Supplementary Figure 1 | Establishing a genome-scale CRISPR activation screen for resistance to T cell cytotoxicity. (**a**) Percent indels generated by different CRISPR knockout (KO) sgRNAs targeting *CTAG1A/B* genes that encode for the NY-ESO-1 antigen. $N = 3$. (**b-c**) Cell survival of NY-ESO-1^+ and HLA-$A2^+$ A375 melanoma cells with different *CTAG1A/B* KO sgRNAs that were co-cultured with T cells expressing the NY-ESO-1 T cell receptor (ESO T cells) relative to cells that have not been exposed to T cells (**b**) or cells co-cultured with unmodified T cells (**c**). $N = 8$. NT, non-targeting. Percent survival at different effector to target (E:T) ratios were measured. Two-tailed *t*-tests with adjustments for multiple comparisons were performed. (**d-k**) MAGeCK analysis results of the acute and chronic exposure screens showing normalized sgRNA counts as counts per million (CPM; **d-e**), sgRNA enrichment in the ESO T cell condition relative to control (**f-g**), gene enrichment determined by the average sgRNA enrichment (**h-i**), and MAGeCK analysis *P*-values (**j-k**). The two most enriched genes from each screening strategy are highlighted in red. All values are mean ± s.e.m. ns, not significant. Source data are provided in Source Data 6 and Supplementary Data 1.

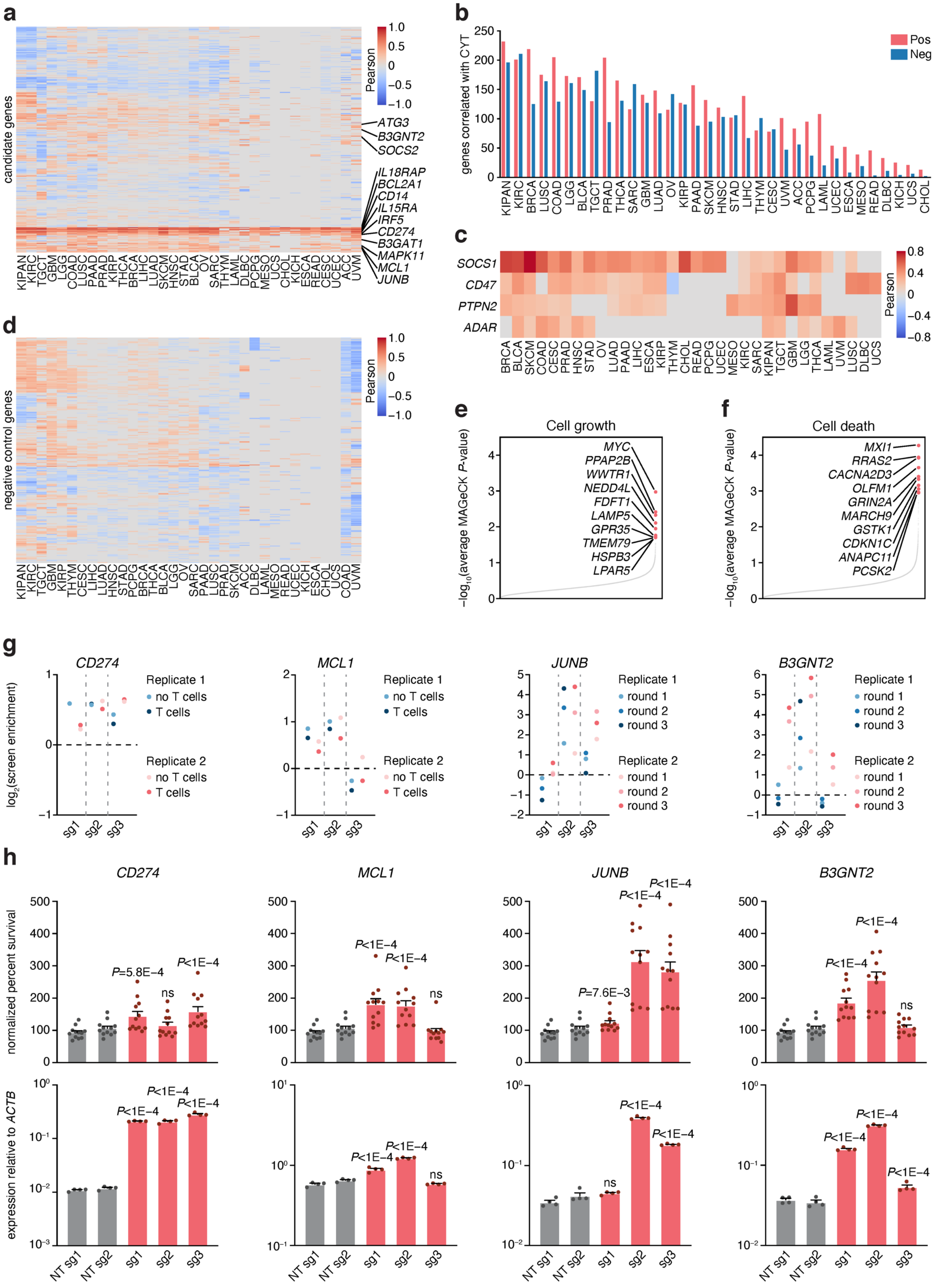
a
candidate genes
Pearson
1.0
0.5
0
−0.5
−1.0
ATG3
B3GNT2
SOCS2
IL18RAP
BCL2A1
CD14
IL15RA
IRF5
CD274
B3GAT1
MAPK11
MCL1
JUNB
b
genes correlated with CYT
Pos
Neg
c
SOCS1
CD47
PTPN2
ADAR
Pearson
d
negative control genes
e
Cell growth
−log10(average MAGeCK P-value)
MYC
PPAP2B
WWTR1
NEDD4L
FDFT1
LAMP5
GPR35
TMEM79
HSPB3
LPAR5
f
Cell death
MXI1
RRAS2
CACNA2D3
OLFM1
GRIN2A
MARCH9
GSTK1
CDKN1C
ANAPC11
PCSK2
g
log2(screen enrichment)
CD274
MCL1
JUNB
B3GNT2
Replicate 1
no T cells
T cells
Replicate 2
round 1
round 2
round 3
sg1
sg2
sg3
h
normalized percent survival
expression relative to ACTB
P=5.8E−4
P<1E−4
P=7.6E−3
ns
NT sg1
NT sg2

Supplementary Figure 2 | Validation of four candidate genes for resistance to T cell cytotoxicity. (**a**) Heatmap showing Pearson's correlation between cytolytic activity and expression of the top 576 candidate genes across patient tumors from TCGA. Only significant (FDR < 0.05) correlations are shown. (**b**) Number of candidate genes with significant (FDR < 0.05) positive or negative Pearson's correlation across patient tumors from TCGA. Out of 34 tumor types, 27 had more genes with positive correlation than negative correlation. (**c-d**) Heatmaps showing significant Pearson's correlations between cytolytic activity and expression of four positive control genes known to promote resistance upon overexpression (**c**), or 291 negative control housekeeping genes (**d**) across patient tumors from TCGA. (**e-f**) Average MAGeCK analysis *P*-values of genes that promote (**e**) or reduce (**f**) A375 cell fitness determined by comparing the distribution of sgRNAs in the CRISPRa screen control conditions to the initial sgRNA library. (**g**) Enrichment of CRISPR activation sgRNAs targeting each candidate gene for different screening biological replicates (bioreps). (**h**) T cell cytotoxicity resistance (n = 12) and transcriptional upregulation (n = 4) in A375 cells upon CRISPR activation of candidate genes. NT, non-targeting. All values are mean ± s.e.m. ns, not significant. Two-tailed t-tests were performed. Source data are provided in Source Data 7, Supplementary Data 1, and Supplementary Data 5.

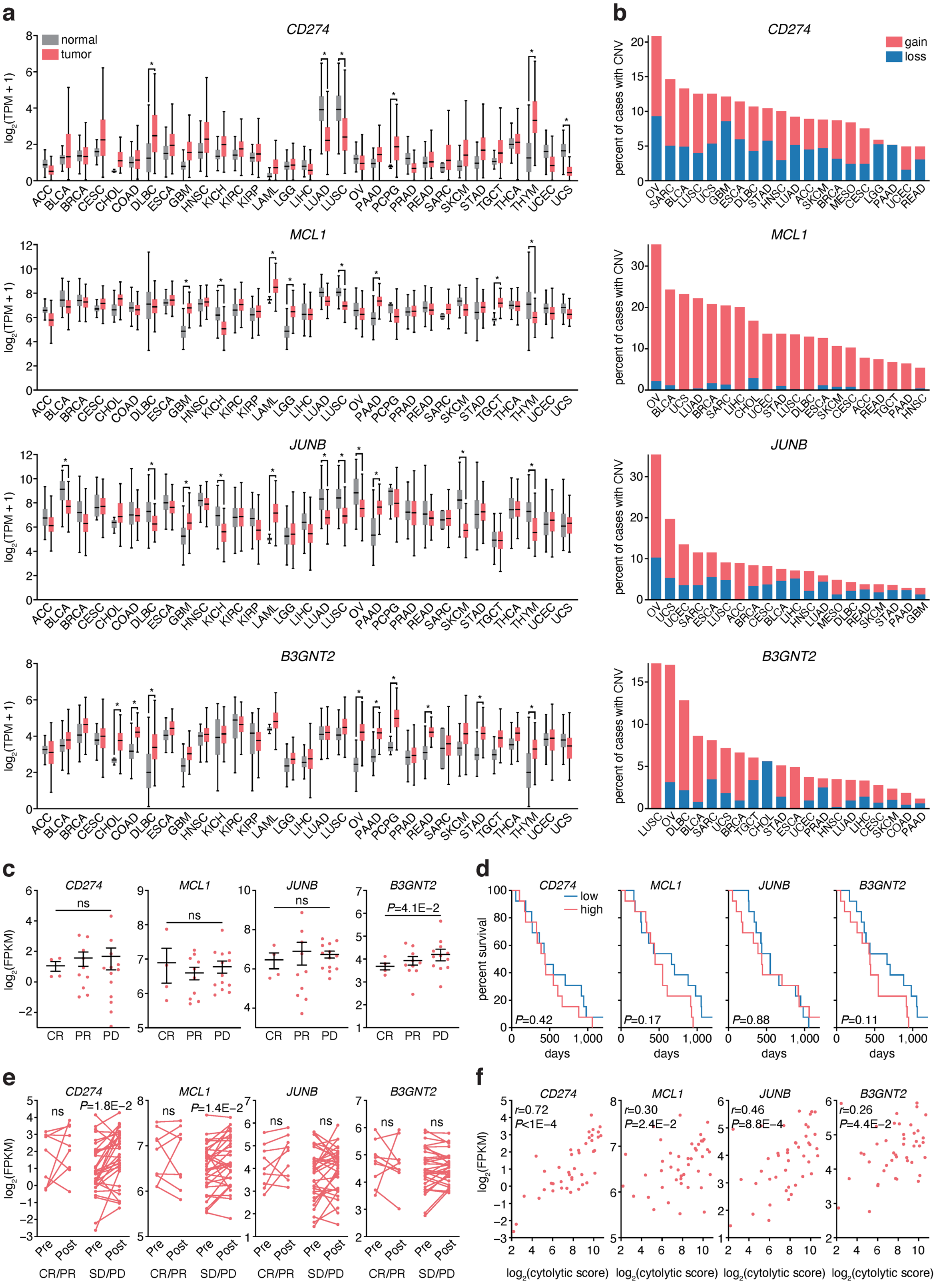
a
CD274
normal
tumor
log2(TPM + 1)
MCL1
JUNB
B3GNT2
ACC BLCA BRCA CESC CHOL COAD DLBC ESCA GBM HNSC KICH KIRC KIRP LAML LGG LIHC LUAD LUSC OV PAAD PCPG PRAD READ SARC SKCM STAD TGCT THCA THYM UCEC UCS
b
CD274
gain
loss
percent of cases with CNV
MCL1
JUNB
B3GNT2
c
CD274
MCL1
JUNB
B3GNT2
ns
P=4.1E−2
log2(FPKM)
CR PR PD
d
CD274
MCL1
JUNB
B3GNT2
low
high
percent survival
P=0.42
P=0.17
P=0.88
P=0.11
days
e
CD274
MCL1
JUNB
B3GNT2
ns
P=1.8E−2
P=1.4E−2
log2(FPKM)
Pre Post Pre Post
CR/PR SD/PD
f
CD274
MCL1
JUNB
B3GNT2
r=0.72
P<1E−4
r=0.30
P=2.4E−2
r=0.46
P=8.8E−4
r=0.26
P=4.4E−2
log2(FPKM)
log2(cytolytic score)

Supplementary Figure 3 | Relevance of top four candidate genes in patient tumors. (**a**) Box plots showing expression of candidate genes in TCGA datasets for 31 types of human cancers. Tumor types with significantly differentially expressed genes (two-tailed *t*-tests with adjustments for multiple comparisons) between tumor (n = 9,736) and matched normal tissues (n = 8,587) are indicated. *$P < 0.05$. Box plots indicate median (middle line), 25th, 75th percentile (box), and whiskers are defined by 1.5 times the interquartile range (*i.e.*, it is the distance between the upper and lower quartiles). (**b**) Focal copy number variation of candidate genes represented as percent of cases for each type of cancer in TCGA. (**c**) Comparison of candidate gene expression and clinical response to anti-PD-1 treatment using patient data from the Hugo et al. dataset [1]. Mean ± s.e.m. is shown. CR (n = 4), PR (n = 10), PD (n = 13). Two-tailed Mann-Whitney tests were performed. (**d**) Overall survival of patients in the Hugo et al. dataset [1]. Patients were stratified based on high (top 50%) or low (bottom 50%) expression of candidate genes. N = 13 for each expression group. Mantel-Cox log-rank tests were performed. (**e**) Comparison of candidate gene expression before (pre) and after (post) anti-PD-1 treatment using matched patient tumor samples from the Riaz et al. dataset [2]. Data from responders (CR or PR, n = 9 per timepoint) and non-responders (SD or PD, n = 33 per timepoint) were grouped together. Pre- and post-immunotherapy expression levels were compared using two-tailed paired *t*-test. (**f**) Pearson correlation between candidate gene expression and cytolytic score [3] prior to treatment using patient data from Riaz et al. [2]. N = 43. Two-tailed Pearson correlation P values are shown. CR, complete response; PR, partial response; SD, stable disease; PD, progressive disease. Source data are provided in Source Data 8.

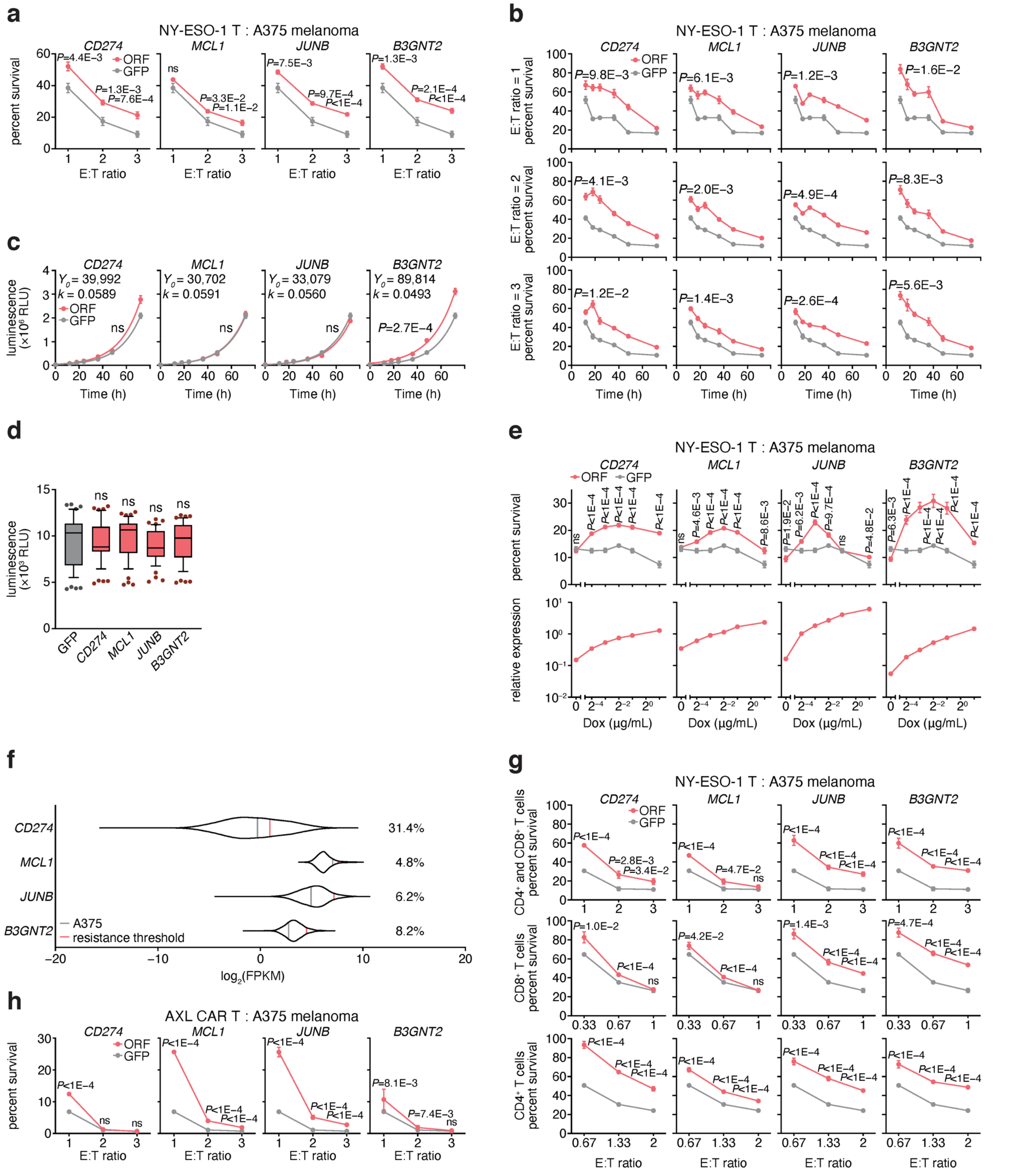

Supplementary Figure 4 | Candidate genes mediate resistance to T cell cytotoxicity across different co-culture conditions. (**a**) Cell survival against ESO T cell-mediated cytotoxicity in A375 cells overexpressing candidate gene ORFs. T cells were derived from donors that were not used in the CRISPRa screen. *N* = 8. (**b**) Cell survival against ESO T cell-mediated cytotoxicity in

A375 cells overexpressing candidate gene ORFs over time. Viability was measured using an alternative assay based on secreted *Gaussia* luciferase. $N = 8$. Repeated measures ANOVA with adjustments for multiple comparisons were performed. (**c**) Growth of A375 cells overexpressing candidate gene ORFs in the absence of ESO T cell co-culture over time measured using secreted *Gaussia* luciferase. Data was fitted using the Malthusian exponential growth $Y(t) = Y_0 e^{kt}$, with t = time, Y = luminescence, k = growth rate. $N = 8$. The growth rates were compared using the extra sum-of-squares F test. (**d**) Box plots showing growth of A375 cells overexpressing candidate gene ORFs in the absence of ESO T cell co-culture measured using a luminescent cell viability assay from 5 independent experiments with $n = 44$. Box plots indicate median (middle line), 25th, 75th percentile (box), and 10th and 90th percentile (whiskers). Two-tailed *t*-tests were performed. (**e**) Dox-induction of candidate genes in A375 cells. Cell survival against ESO T cell cytotoxicity ($n = 8$) and candidate gene expression relative to *ACTB* control ($n = 4$) were measured at different Dox concentrations. (**f**) Expression of candidate genes in 1,019 cell lines from the Cancer Cell Line Encyclopedia [4]. Expression in A375 cells and the expression threshold above which the candidate gene conferred resistance to T cell cytotoxicity in A375 cells are indicated. The percentage of cell lines with gene expression above the resistance threshold is shown. (**g**) Cell survival of A375 cells overexpressing candidate genes against $CD4^+$, $CD8^+$, or $CD4^+$ and $CD8^+$ T cell cytotoxicity. $N = 8$. (**h**) Cell survival of A375 cells overexpressing candidate genes against T cells expressing the AXL chimeric antigen receptor (CAR). $N = 8$. All values are mean ± s.e.m. ns, not significant. Two-tailed *t*-tests with adjustments for multiple comparisons were performed unless otherwise indicated. Source data are provided in Source Data 9.

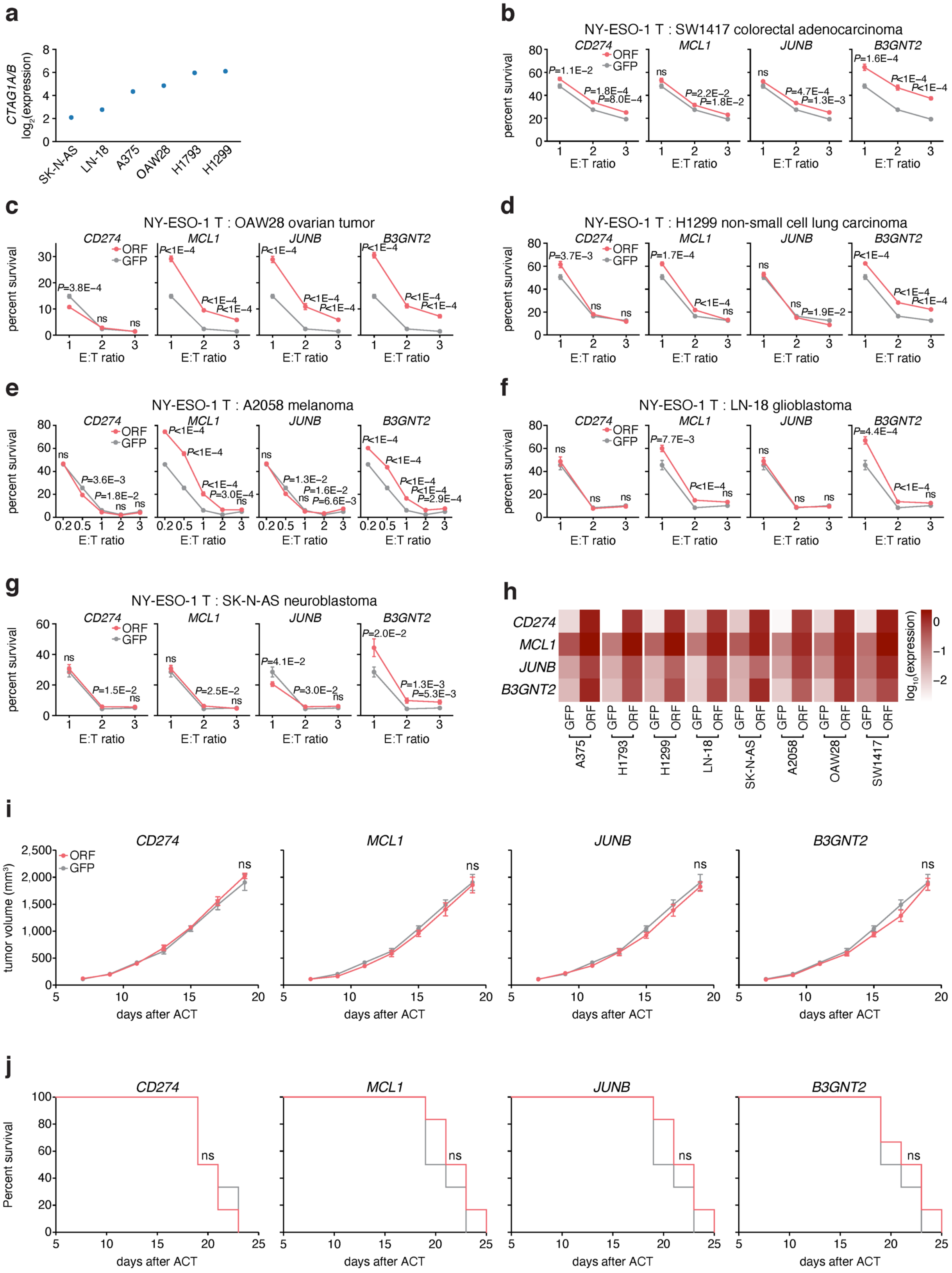
a
b
NY-ESO-1 T : SW1417 colorectal adenocarcinoma
c
NY-ESO-1 T : OAW28 ovarian tumor
d
NY-ESO-1 T : H1299 non-small cell lung carcinoma
e
NY-ESO-1 T : A2058 melanoma
f
NY-ESO-1 T : LN-18 glioblastoma
g
NY-ESO-1 T : SK-N-AS neuroblastoma
h
i
j
CD274
MCL1
JUNB
B3GNT2
ORF
GFP
percent survival
E:T ratio
tumor volume (mm³)
days after ACT
Percent survival
log10(expression)
SK-N-AS
LN-18
A375
OAW28
H1793
H1299
A2058
SW1417

Supplementary Figure 5 | Candidate genes mediate resistance to T cell cytotoxicity in other cell types and *in vivo*. (**a**) Expression level of the NY-ESO-1 antigen represented as the average expression of *CTAG1A* and *CTAG1B* genes. RNA-seq expression data for each cell line was obtained from the Cancer Cell Line Encyclopedia [4]. (**b-g**) Resistance to ESO T cell-mediated cytotoxicity in SW1417 (NY-ESO-1^{-}, HLA-A2^{-}) colorectal adenocarcinoma (**b**), OAW28 (NY-ESO-1^{+}, HLA-A2^{-}) ovarian cystadenocarcinoma (**c**), H1299 (NY-ESO-1^{+}, HLA-A2^{-}) non-small cell lung carcinoma (**d**), A2058 (NY-ESO-1^{-}, HLA-A2^{-}) melanoma (**e**), LN-18 (NY-ESO-1^{+}, HLA-A2^{+}) glioblastoma (**f**), or SK-N-AS (NY-ESO-1^{+}, HLA-A2^{-}) neuroblastoma (**g**) cell lines. Cell lines that did not endogenously express HLA-A2 or NY-ESO-1 were transduced with the respective constructs. $N = 8$ (**b**, **c**, **e**) or 12 (**d**, **f**, **g**). Two-tailed *t*-tests with adjustments for multiple comparisons were performed. (**h**) Heatmap showing average expression ($n = 4$) relative to *ACTB* of candidate genes in various cell lines overexpressing the ORF or GFP. (**i-j**) Tumor growth in control mice that did not receive adoptive cell transfer (ACT). $N = 6$. (**i**) Tumor volume is shown. Two-tailed *t*-tests with adjustments for multiple comparisons were performed. (**j**) Overall survival is shown. Mantel-Cox log-rank tests were performed. All values are mean ± s.e.m. ns, not significant. Source data are provided in Source Data 10.

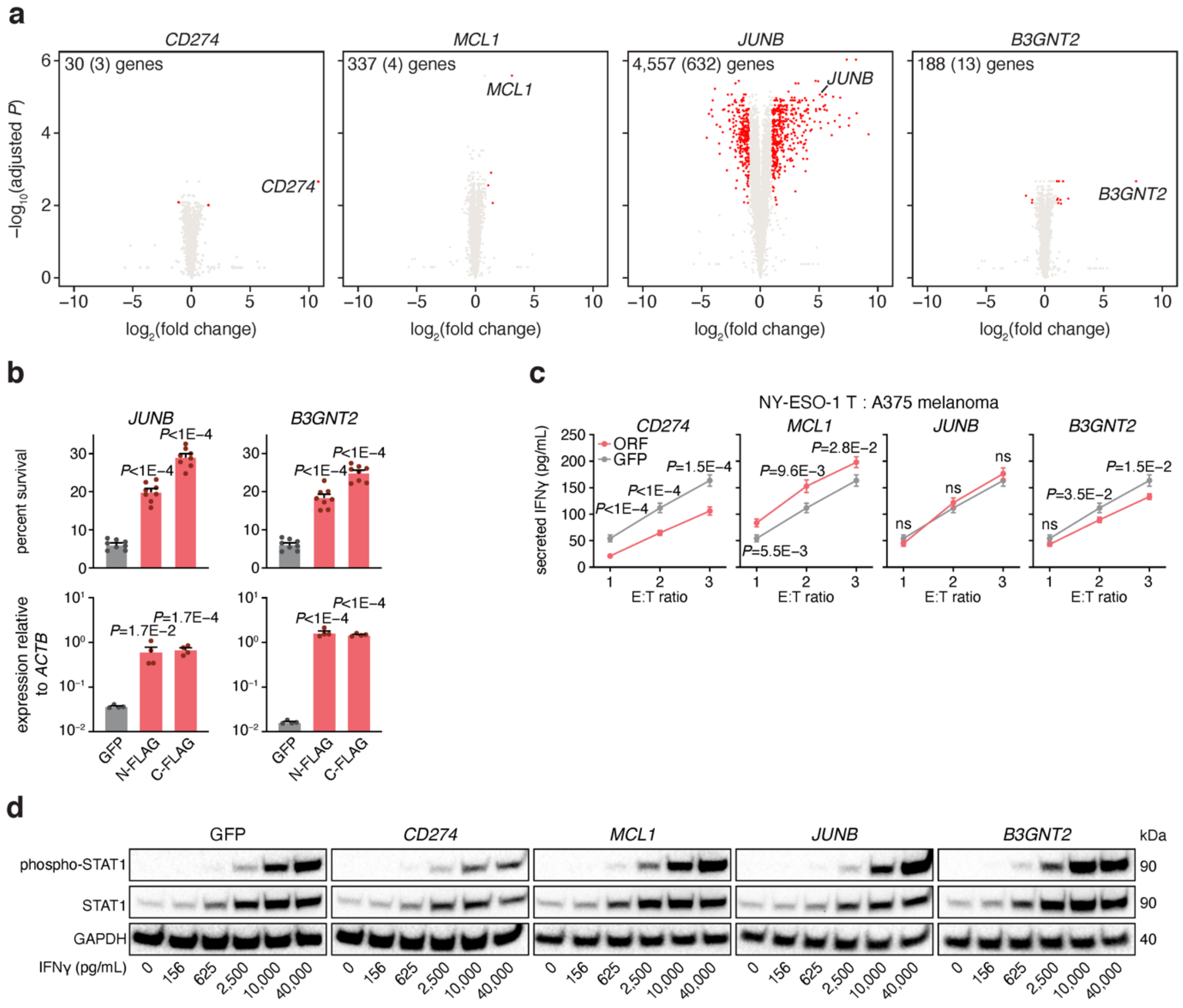

Supplementary Figure 6 | Effect of candidate gene overexpression on the transcriptome and IFNγ pathways. (**a**) Volcano plots showing transcriptome changes measured by RNA-seq in A375 cells overexpressing candidate genes. The number of genes that were significantly differentially expressed with *P*-value ≥ 0.01 FDR correction based on two-tailed *t*-tests are indicated. Out of these genes, those with $|\log_2(fold\ change)| \geq 1$ are shown as red dots and the number of genes is indicated in parentheses. $N = 3$. (**b**) T cell cytotoxicity resistance ($n = 8$) and transcriptional upregulation ($n = 4$) in A375 cells overexpressing N- or C-terminal FLAG tagged (N-FLAG or C-FLAG respectively) *JUNB* or *B3GNT2*. Two-tailed *t*-tests were performed. (**c**) IFNγ measured by ELISA in cell culture media of ESO T cells co-cultured with A375 cells overexpressing candidate genes. $N = 16$. Two-tailed *t*-tests with adjustments for multiple comparisons were performed. (**d**) Western blots of phosphorylated or total STAT1 in A375 cells overexpressing candidate genes that have been exposed to different concentrations of IFNγ. Data is representative of two

independent experiments. All values are mean ± s.e.m. ns, not significant. Source data are provided in Source Data 11 and Supplementary Data 6.

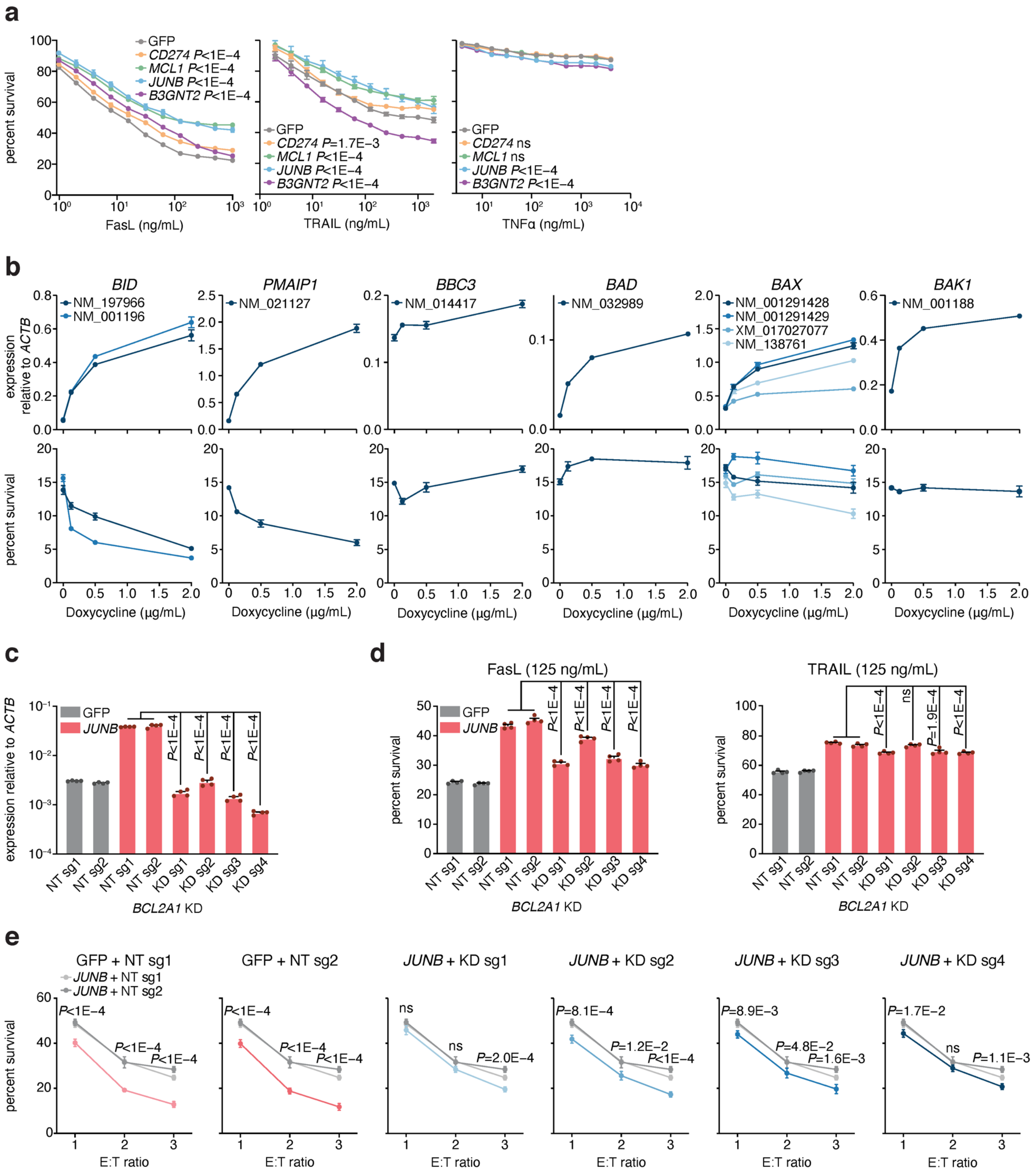

Supplementary Figure 7 | *MCL1* and *JUNB* mediate survival by resisting FasL- and TRAIL-induced cytotoxicity. (**a**) Cell survival of A375 cells overexpressing candidate genes against different concentrations of FasL, TRAIL, or TNFα. N = 4. Repeated measures ANOVA with adjustments for multiple comparisons were performed. (**b**) Dox-induction of different RefSeq isoforms of genes in the mitochondrial apoptosis pathway in A375 cells overexpressing *MCL1*.

Cell survival against ESO T cell-induced cytotoxicity (n = 8) and expression (n = 4) of *MCL1* interaction partners were measured at different Dox concentrations. (**c**) Expression of *BCL2A1* in A375 cells overexpressing *JUNB* or *B3GNT2* with *BCL2A1* knocked down. N = 4. KD, knockdown. NT, non-targeting. Two-tailed t-tests were performed. (**d**) Cell survival against 125 ng/µL of FasL- or TRAIL-induced cell death in A375 cells overexpressing *JUNB* or GFP with *BCL2A1* knocked down. N = 4. Two-tailed t-tests were performed. (**e**) Cell survival against ESO T cells in A375 cells overexpressing *JUNB* or GFP with *BCL2A1* knocked down. N = 8. Two-tailed t-tests with adjustments for multiple comparisons were performed. All values are mean ± s.e.m. ns, not significant. Source data are provided in Source Data 12.

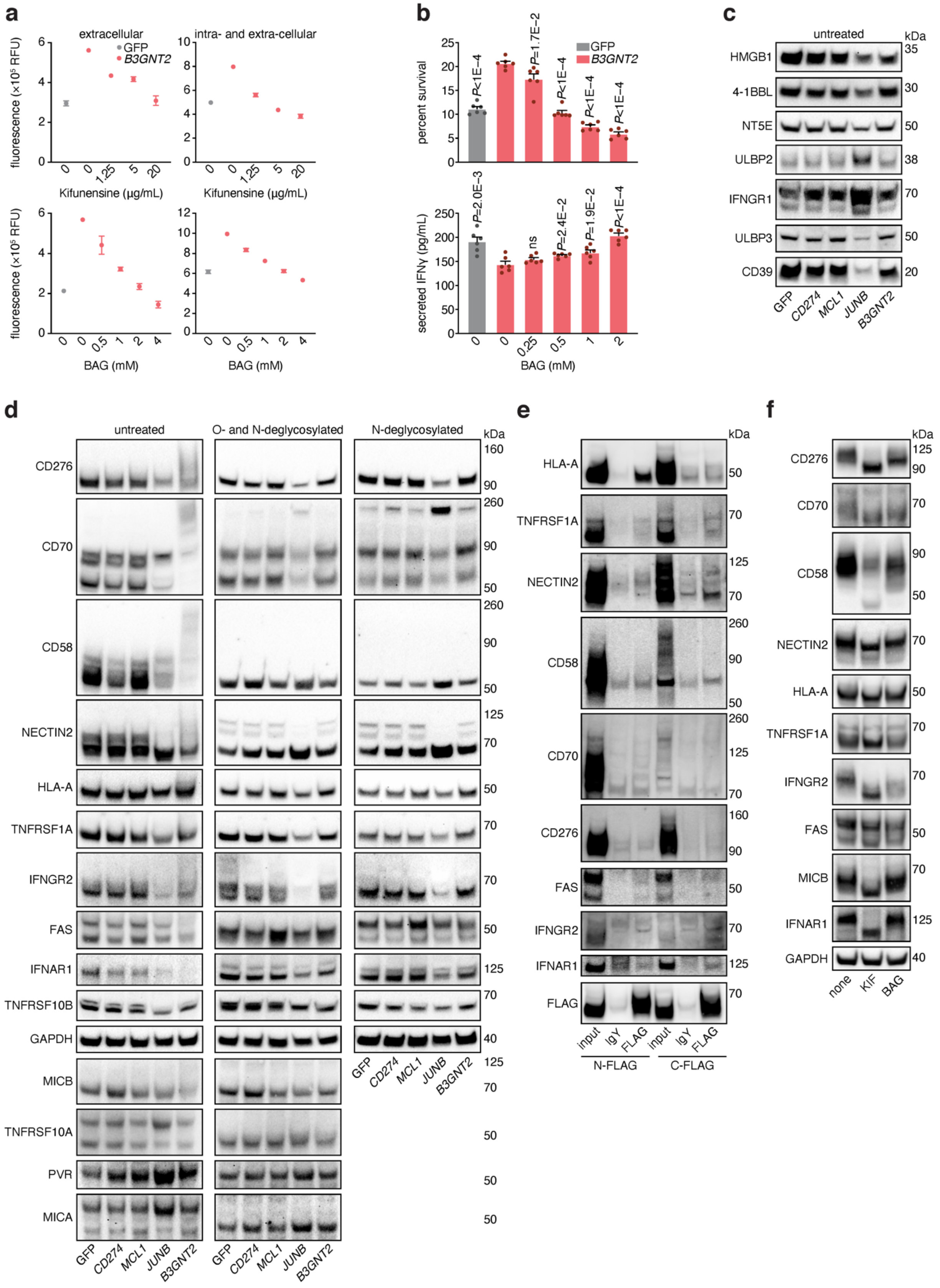
a
extracellular
GFP
B3GNT2
intra- and extra-cellular
fluorescence (×10⁵ RFU)
Kifunensine (µg/mL)
0 0 1.25 5 20
BAG (mM)
0 0 0.5 1 2 4
b
percent survival
GFP
B3GNT2
P<1E−4
P=1.7E−2
P<1E−4
P<1E−4
P<1E−4
secreted IFNγ (pg/mL)
P=2.0E−3
ns
P=2.4E−2
P=1.9E−2
P<1E−4
BAG (mM)
0 0 0.25 0.5 1 2
c
untreated
kDa
HMGB1 35
4-1BBL 30
NT5E 50
ULBP2 38
IFNGR1 70
ULBP3 50
CD39 20
GFP
CD274
MCL1
JUNB
B3GNT2
d
untreated
O- and N-deglycosylated
N-deglycosylated
kDa
CD276 160 90
CD70 260 90 50
CD58 260 90 50
NECTIN2 125 70
HLA-A 50
TNFRSF1A 70
IFNGR2 70
FAS 50
IFNAR1 125
TNFRSF10B 70
GAPDH 40
MICB 125 70
TNFRSF10A 50
PVR 50
MICA 50
GFP
CD274
MCL1
JUNB
B3GNT2
e
kDa
HLA-A 50
TNFRSF1A 70
NECTIN2 125 70
CD58 260 90 50
CD70 260 125 70
CD276 160 90
FAS 50
IFNGR2 70
IFNAR1 125
FLAG 70
input
IgY
FLAG
N-FLAG
C-FLAG
f
kDa
CD276 125 90
CD70 70
CD58 90 50
NECTIN2 70
HLA-A 50
TNFRSF1A 70
IFNGR2 70
FAS 50
MICB 70
IFNAR1 125
GAPDH 40
none
KIF
BAG

Supplementary Figure 8 | *B3GNT2* adds poly-LacNAc to tumor ligands and receptors to promote tumor immune evasion. (**a**) Intra- and extra-cellular poly-LacNAc measured by tomato lectin staining in A375 cells overexpressing *B3GNT2* or GFP that were treated with different concentrations of kifunensine or benzyl-2-acetamido-2-deoxy-α-D-galactopyranoside (BAG) glycosylation inhibitors. $N = 2$. (**b**) Cell survival against T cell cytotoxicity (top) and T cell IFNγ secretion (bottom) in A375 cells overexpressing *B3GNT2* or GFP that have been treated with different concentrations of BAG at E:T ratio of 3. BAG was used to pretreat A375 cells and was present during co-culture. $N = 6$. Two-tailed *t*-tests were performed. (**c-d**) Western blots of different tumor ligands and receptors that interact with T cells in A375 cells overexpressing candidate genes. For a subset of the ligands and receptors that were potentially glycosylated (**d**), enzymatic deglycosylation was performed to confirm presence of *N*- or *O*-glycosylation. Data is representative of two independent experiments. (**e**) Co-IP of N- or C-terminal FLAG tagged (N-FLAG or C-FLAG) *B3GNT2* followed by Western blot for different *B3GNT2* target proteins. 2% of the input and IgY IP controls are shown. Data is representative of two independent experiments. (**f**) Western blots of tumor cell surface ligands and receptors that interact with T cells in SW1417 colorectal adenocarcinoma cells. Cells were treated with kifunensine (KIF) or BAG to inhibit *N*- or *O*- glycosylation respectively. Data is representative of two independent experiments. All values are mean ± s.e.m. ns, not significant. Source data are provided in Source Data 13.

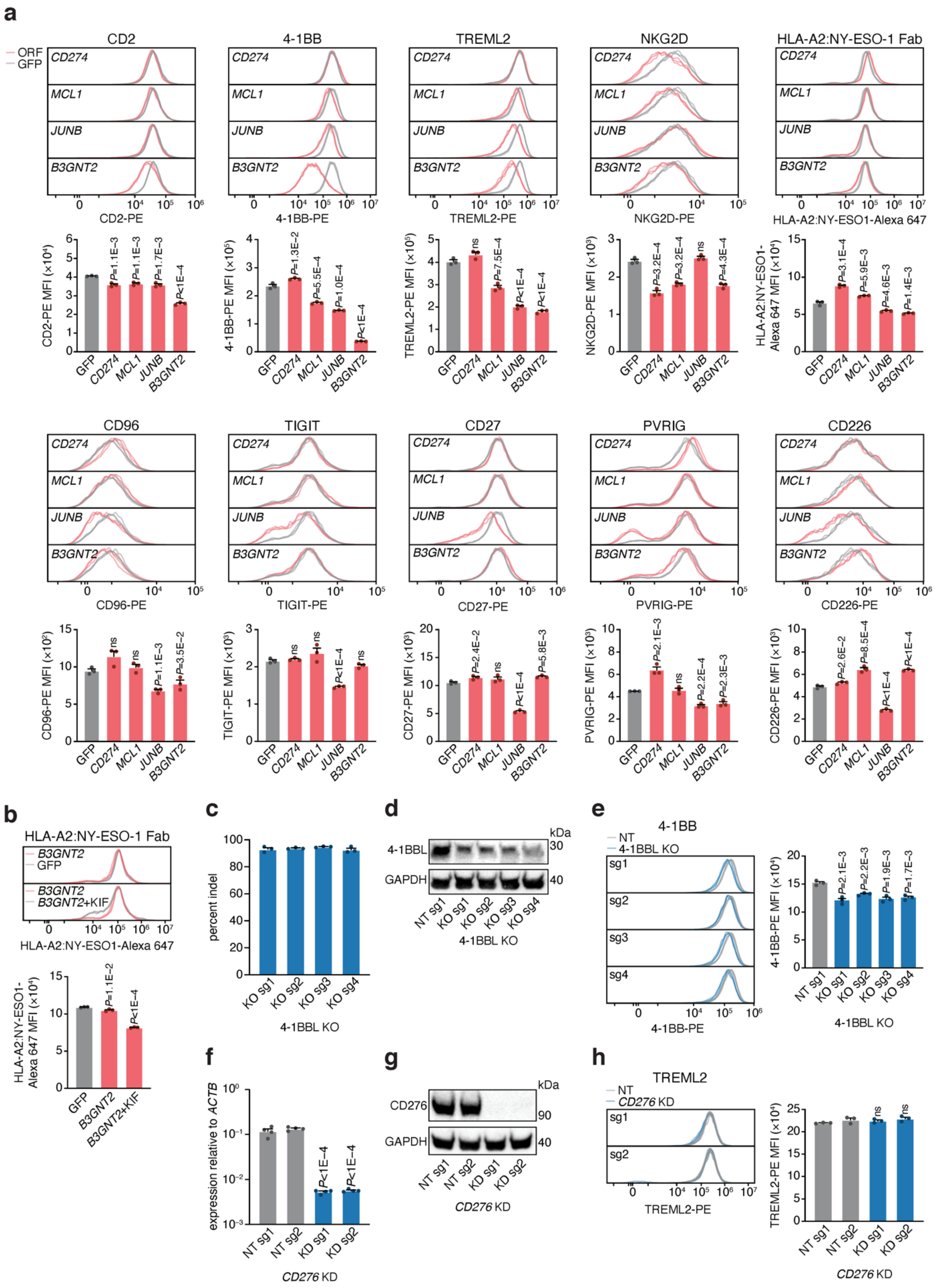
a
CD2
4-1BB
TREML2
NKG2D
HLA-A2:NY-ESO-1 Fab
ORF
GFP
CD274
MCL1
JUNB
B3GNT2
CD2-PE
4-1BB-PE
TREML2-PE
NKG2D-PE
HLA-A2:NY-ESO1-Alexa 647
CD2-PE MFI (×10⁴)
4-1BB-PE MFI (×10⁵)
TREML2-PE MFI (×10⁵)
NKG2D-PE MFI (×10³)
HLA-A2:NY-ESO1-Alexa 647 MFI (×10⁴)
GFP
P=1.1E−3
P=1.1E−3
P=1.7E−3
P<1E−4
P=1.3E−2
P=5.5E−4
P=1.0E−4
P<1E−4
ns
P=7.5E−4
P<1E−4
P<1E−4
P=3.2E−4
P=3.2E−4
ns
P=4.3E−4
P=3.1E−4
P=5.9E−3
P=4.6E−3
P=1.4E−3
CD96
TIGIT
CD27
PVRIG
CD226
CD96-PE
TIGIT-PE
CD27-PE
PVRIG-PE
CD226-PE
CD96-PE MFI (×10²)
TIGIT-PE MFI (×10³)
CD27-PE MFI (×10³)
PVRIG-PE MFI (×10³)
CD226-PE MFI (×10³)
ns
ns
P=1.1E−3
P=3.5E−2
ns
ns
P<1E−4
ns
P=2.4E−2
ns
P<1E−4
P=5.8E−3
P=2.1E−3
ns
P=2.2E−4
P=2.3E−3
P=2.6E−2
P=8.5E−4
P<1E−4
P<1E−4
b
HLA-A2:NY-ESO-1 Fab
B3GNT2
GFP
B3GNT2+KIF
HLA-A2:NY-ESO1-Alexa 647
HLA-A2:NY-ESO1-Alexa 647 MFI (×10⁴)
P=1.1E−2
P<1E−4
c
percent indel
KO sg1
KO sg2
KO sg3
KO sg4
4-1BBL KO
d
kDa
4-1BBL
GAPDH
30
40
NT sg1
KO sg1
KO sg2
KO sg3
KO sg4
4-1BBL KO
e
4-1BB
NT
4-1BBL KO
sg1
sg2
sg3
sg4
4-1BB-PE
4-1BB-PE MFI (×10⁴)
P=2.1E−3
P=2.2E−3
P=1.9E−3
P=1.7E−3
4-1BBL KO
f
expression relative to ACTB
P<1E−4
P<1E−4
NT sg1
NT sg2
KD sg1
KD sg2
CD276 KD
g
kDa
CD276
GAPDH
90
40
NT sg1
NT sg2
KD sg1
KD sg2
CD276 KD
h
TREML2
NT
CD276 KD
sg1
sg2
TREML2-PE
TREML2-PE MFI (×10⁴)
ns
ns
CD276 KD

Supplementary Figure 9 | *B3GNT2* overexpression disrupts binding of T cell ligands and receptors to tumor cells. (**a**) Histograms (top) and corresponding median fluorescence intensity (MFI; bottom) showing binding of various T cell ligands or receptor proteins to A375 cells overexpressing candidate genes measured by flow cytometry. $N = 3$. (**b**) Histograms (top) and corresponding MFI (bottom) showing binding of an antibody specific for HLA-A2:NY-ESO-1 to A375 cells overexpressing *B3GNT2* or GFP that have been treated with kifunensine (KIF). $N = 3$. (**c**) Percent indels generated by different CRISPR knockout (KO) sgRNAs targeting 4-1BBL (*TNFSF9*) in A375 cells. $N = 3$. (**d**) Protein expression of 4-1BBL after CRISPR KO. Data is representative of two independent experiments. (**e**) Histograms (left) and corresponding MFI (right) showing binding of 4-1BB to A375 cells with different KO sgRNAs targeting 4-1BBL. $N = 3$. (**f**) *CD276* expression in A375 cells with different CRISPR knockdown (KD) sgRNAs. $N = 4$. (**g**) Protein expression of CD276 after CRISPR KD. Data is representative of two independent experiments. (**h**) Histograms (left) and corresponding MFI (right) showing binding of TREML2 to A375 cells with different KD sgRNAs targeting *CD276*. $N = 3$. All values are mean ± s.e.m. NT, non-targeting; ns, not significant. Two-tailed *t*-tests were performed. Source data are provided in Source Data 14.

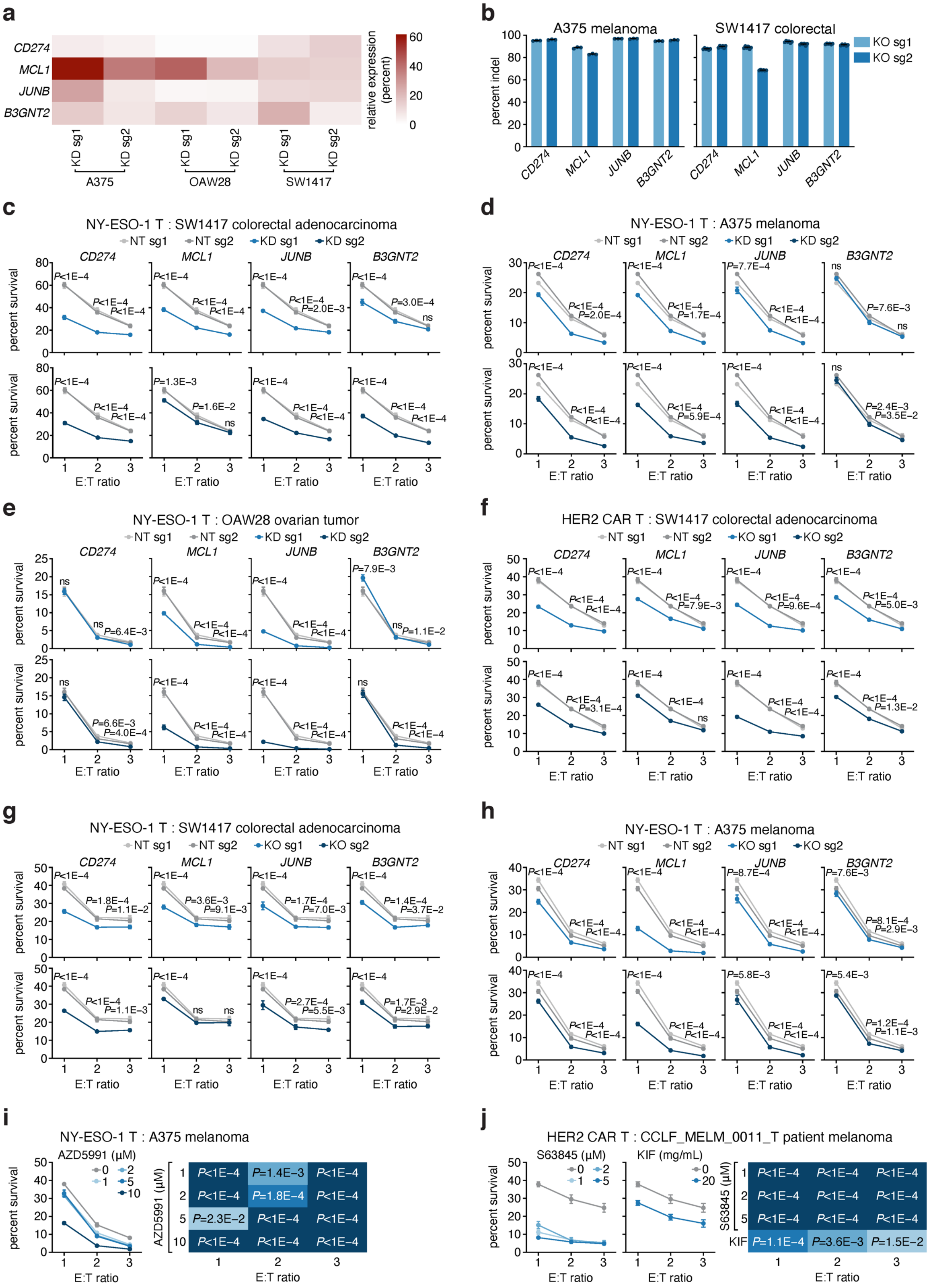
a
CD274
MCL1
JUNB
B3GNT2
KD sg1
KD sg2
A375
OAW28
SW1417
relative expression (percent)
60
40
20
0
b
A375 melanoma
SW1417 colorectal
KO sg1
KO sg2
percent indel
c
NY-ESO-1 T : SW1417 colorectal adenocarcinoma
NT sg1
NT sg2
KD sg1
KD sg2
percent survival
E:T ratio
d
NY-ESO-1 T : A375 melanoma
e
NY-ESO-1 T : OAW28 ovarian tumor
f
HER2 CAR T : SW1417 colorectal adenocarcinoma
KO sg1
KO sg2
g
NY-ESO-1 T : SW1417 colorectal adenocarcinoma
h
NY-ESO-1 T : A375 melanoma
i
NY-ESO-1 T : A375 melanoma
AZD5991 (μM)
0
1
2
5
10
AZD5991 (μM)
1 P<1E–4 P=1.4E–3 P<1E–4
2 P<1E–4 P=1.8E–4 P<1E–4
5 P=2.3E–2 P<1E–4 P<1E–4
10 P<1E–4 P<1E–4 P<1E–4
j
HER2 CAR T : CCLF_MELM_0011_T patient melanoma
S63845 (μM)
KIF (mg/mL)
20
S63845 (μM)
1 P<1E–4 P<1E–4 P<1E–4
2 P<1E–4 P<1E–4 P<1E–4
5 P<1E–4 P<1E–4 P<1E–4
KIF P=1.1E–4 P=3.6E–3 P=1.5E–2

Supplementary Figure 10 | Inhibition of candidate genes increases susceptibility of tumors to T cell killing upon knockdown. (**a**) Heatmap showing expression of candidate genes in different cell lines transduced with CRISPR knockdown (KD) sgRNAs relative to non-targeting (NT) sgRNAs. $N = 4$. (**b**) Percent indels generated by different CRISPR knockout (KO) sgRNAs targeting candidate genes in A375 melanoma ($N = 3$) and SW1417 colorectal adenocarcinoma cells ($N = 6$). (**c-h**) Cell survival against T cell-mediated cytotoxicity with different candidate genes knocked down (**c-e**) or knocked out (**f-h**). Cell lines that did not endogenously express HLA-A2 or NY-ESO-1 were transduced with the respective constructs. $N = 8$. (**c**) SW1417 (NY-ESO-1^{-}, HLA-$A2^{-}$) colorectal adenocarcinoma against ESO T cells. (**d**) A375 (NY-ESO-1^{+}, HLA-$A2^{+}$) melanoma against ESO T cells. (**e**) OAW28 (NY-ESO-1^{+}, HLA-$A2^{-}$) ovarian cystadenocarcinoma against ESO T cells. (**f**) SW1417 ($HER2^{+}$) colorectal adenocarcinoma against HER2 CAR T cells. (**g**) SW1417 (NY-ESO-1^{-}, HLA-$A2^{-}$) colorectal adenocarcinoma against ESO T cells. (**h**) A375 (NY-ESO-1^{+}, HLA-$A2^{+}$) melanoma against ESO T cells. (**i-j**) Cell survival against T cell cytotoxicity in tumor cells treated with small molecule inhibitors targeting MCL1 (S63845 and AZD5991) or B3GNT2 (kifunensine). Heatmaps show statistical analysis results for each condition. $N = 8$. (**i**) A375 (NY-ESO-1^{+}, HLA-$A2^{+}$) melanoma against ESO T cells. (**j**) CCLF_MELM_0011_T ($HER2^{+}$) primary patient-derived melanoma model against HER2 CAR T cells. All values are mean ± s.e.m. ns, not significant. Two-tailed *t*-tests with adjustments for multiple comparisons were performed. Source data are provided in Source Data 15.

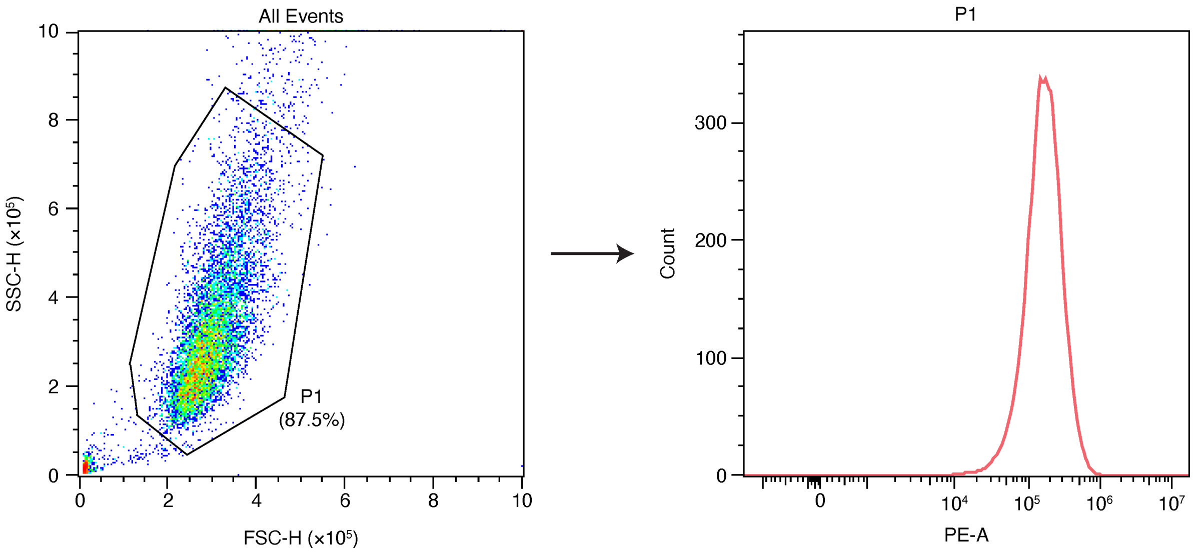

Supplementary Figure 11 | Example flow cytometry gating strategy. Cells were gated on forward scatter (FSC) and side scatter (SSC) signals to discard debris. After eliminating debris, median fluorescence intensity was measured on P1. Example gating strategy is representative of all flow cytometry data presented.

Supplementary Tables

Supplementary Table 1 | List of sgRNAs and primers used in the study. (**a**) sgRNA ID, perturbation, target sequences, and target genes. (**b**) Primers for indel amplification and quantification.

**Target gene**	**sgRNA ID**	**Perturbation**	**Target sequence (5' to 3')**
CTAG1A/B	sg1	knockout	GCGGGGTCCGCATGGCGGCG
CTAG1A/B	sg2	knockout	CAGAATACAACTCAAGCAGG
CTAG1A/B	sg3	knockout	GAATGGATGCTGCAGATGCG
CD274	sg1	activation	CTGACCTTCGGTGAAATCGG
CD274	sg2	activation	TCAGTTTAGGTATCTAGTGT
CD274	sg3	activation	CTATACACAGCTTTATTCCT
MCL1	sg1	activation	CATGGAAAGAGCTCGAGCCC
MCL1	sg2	activation	CACTCAGAGCCTCCGAAGAC
MCL1	sg3	activation	CGGAGCCGCCGTTACGTAAC
JUNB	sg1	activation	CCCCTCCTCGAGCGTGGGGA
JUNB	sg2	activation	AGGCGGCTCGCGTCACTGTC
JUNB	sg3	activation	GCGCGTGTCCTTGTAAACAG
B3GNT2	sg1	activation	GCCGCAGGGAGCGCGGGCCC
B3GNT2	sg2	activation	GTGGGTCCTGGTACCGGGTG
B3GNT2	sg3	activation	CGGAACCCTCCCAAAACTTG
CD274	sg1	knockdown	AGCAGCTGGCGCGTCCCGCG
CD274	sg2	knockdown	TCGGGAAGCTGCGCAGAACT
MCL1	sg1	knockdown	AGCTTCCGGAGGGTTGCGCA
MCL1	sg2	knockdown	CCTTTATCACGGTTTTAGGG
JUNB	sg1	knockdown	CTGGGACCTTGAGAGCGGCC
JUNB	sg2	knockdown	TATCGCGCCAGAGAGGGCGA
B3GNT2	sg1	knockdown	CTGCGCCTCACTCCAGGCTC
B3GNT2	sg2	knockdown	GGAGTGAGGCGCAGCGGCAG

BCL2A1	sg1	knockdown	TACGCACGAAAGTGACTAGG
BCL2A1	sg2	knockdown	ACATGATGATACATGGAGGC
BCL2A1	sg3	knockdown	GGCTCACCTTGAAGCTGTTG
BCL2A1	sg4	knockdown	TCAAGACTTTGCTCTCCACC
CD276	sg1	knockdown	GCGGCTCCGGTGCGTCCCTG
CD276	sg2	knockdown	GCGTCCCTGAGTCCCAGAGT
CD274	sg1	knockout	ACATGTCAGTTCATGTTCAG
CD274	sg2	knockout	GGTTCCCAAGGACCTATATG
MCL1	sg1	knockout	AGTCGCTGGAGATTATCTCT
MCL1	sg2	knockout	CCAAAAGTCGCCCTCCCGGG
JUNB	sg1	knockout	CCGGAGTCTCAAAGCGCCTG
JUNB	sg2	knockout	GGGTAAAAGTACTGTCCCGG
B3GNT2	sg1	knockout	CAACGCAGGGAACCAAACGG
B3GNT2	sg2	knockout	GGTTCCAGTATGCCTCGGGA
TNFSF9	sg1	knockout	CCCATCGATCAGCAGAACTG
TNFSF9	sg2	knockout	GCCAGCCCGAGACTCCGCGA
TNFSF9	sg3	knockout	GGGGGGCCTGAGCTACAAAG
TNFSF9	sg4	knockout	TCAACTAGAGCTGCGGCGCG
Nontargeting	sg1		CTGAAAAAGGAAGGAGTTGA
Nontargeting	sg2		AAGATGAAAGGAAAGGCGTT

Supplementary Table 2 | Primers for indel amplification and quantification.

**Target gene**	**sgRNA ID**	**Primer**	**Sequence (5' to 3')**
CTAG1A/B	sg1	Fwd	CTTTCCCTACACGACGCTCTTCCGATCTGGGCAGCAAGGGCCTC
CTAG1A/B	sg1	Rev	GACTGGAGTTCAGACGTGTGCTCTTCCGATCTGCTCTCCGGCCCCCT
CTAG1A/B	sg2	Fwd	CTTTCCCTACACGACGCTCTTCCGATCTTCAGGGCTGAATGGATGCTG

CTAG1A/B	sg2	Rev	GACTGGAGTTCAGACGTGTGCTCTTCCGATCT TGCCCTCCCCATCTCCC
CTAG1A/B	sg3	Fwd	CTTTCCCTACACGACGCTCTTCCGATCTGGGC AGCAAGGGCCTC
CTAG1A/B	sg3	Rev	GACTGGAGTTCAGACGTGTGCTCTTCCGATCT GCCCCCACCTCGCCA
CD274	sg1	Fwd	CTTTCCCTACACGACGCTCTTCCGATCTTGTT TATGTCCTAGCCCCATAC
CD274	sg1	Rev	GACTGGAGTTCAGACGTGTGCTCTTCCGATCT CTTGATGGTCACTGCTTGTCC
CD274	sg2	Fwd	CTTTCCCTACACGACGCTCTTCCGATCTAAAC GCTGTGCCAATTTTGTAAATG
CD274	sg2	Rev	GACTGGAGTTCAGACGTGTGCTCTTCCGATCT GACAATTAGTGCAGCCAGGTCTA
MCL1	sg1	Fwd	CTTTCCCTACACGACGCTCTTCCGATCTGGAG TTGGTCGGGGAATCTG
MCL1	sg1	Rev	GACTGGAGTTCAGACGTGTGCTCTTCCGATCT CAACCCGTCGTAAGGTCTCC
MCL1	sg2	Fwd	CTTTCCCTACACGACGCTCTTCCGATCTAAAA GAAACGCGGTAATCGGAC
MCL1	sg2	Rev	GACTGGAGTTCAGACGTGTGCTCTTCCGATCT GCGCTTCCGCCAATCAC
JUNB	sg1	Fwd	CTTTCCCTACACGACGCTCTTCCGATCTGGCC TCTCTCTACACGACTAC
JUNB	sg1	Rev	GACTGGAGTTCAGACGTGTGCTCTTCCGATCT CAGCTCCGAAGAGGCGAG
JUNB	sg2	Fwd	CTTTCCCTACACGACGCTCTTCCGATCTATTG TCCCCAACAGCAACGG
JUNB	sg2	Rev	GACTGGAGTTCAGACGTGTGCTCTTCCGATCT TTGTGCAGATCGTCCAGGGC

B3GNT2	sg1	Fwd	CTTTCCCTACACGACGCTCTTCCGATCTTGCTGGCGATTAAGTCCCTC
B3GNT2	sg1	Rev	GACTGGAGTTCAGACGTGTGCTCTTCCGATCTATCTGAAAGGTCGGGGTGGT
B3GNT2	sg2	Fwd	CTTTCCCTACACGACGCTCTTCCGATCTTCTCCAAAAGCAGTAGCCAAG
B3GNT2	sg2	Rev	GACTGGAGTTCAGACGTGTGCTCTTCCGATCTTATATTGGAGAGCCTGCCCG
TNFSF9	sg1	Fwd	CTTTCCCTACACGACGCTCTTCCGATCTTCTTTTCTCCCAGGGCTGC
TNFSF9	sg1	Rev	GACTGGAGTTCAGACGTGTGCTCTTCCGATCTCTTTGTAGCTCAGGCCCCC
TNFSF9	sg2	Fwd	CTTTCCCTACACGACGCTCTTCCGATCTTTCCTCGCCTGCCCCT
TNFSF9	sg2	Rev	GACTGGAGTTCAGACGTGTGCTCTTCCGATCTGCCGCAGGTCCAAGAGG
TNFSF9	sg3	Fwd	CTTTCCCTACACGACGCTCTTCCGATCTACAAAGAGGACACGAAGGAGC
TNFSF9	sg3	Rev	GACTGGAGTTCAGACGTGTGCTCTTCCGATCTCCCCAGCAGCAGAGCG
TNFSF9	sg4	Fwd	CTTTCCCTACACGACGCTCTTCCGATCTTTTCCTCCCACAGTTCTGCTGAT
TNFSF9	sg4	Rev	GACTGGAGTTCAGACGTGTGCTCTTCCGATCTCGCAGCTCTAGTTGAAAGAAGACA

References

1 Hugo, W. *et al.* Genomic and Transcriptomic Features of Response to Anti-PD-1 Therapy in Metastatic Melanoma. *Cell* **165**, 35-44, doi:10.1016/j.cell.2016.02.065 (2016).

2 Riaz, N. *et al.* Tumor and Microenvironment Evolution during Immunotherapy with Nivolumab. *Cell* **171**, 934-949 e916, doi:10.1016/j.cell.2017.09.028 (2017).

3 Rooney, M. S., Shukla, S. A., Wu, C. J., Getz, G. & Hacohen, N. Molecular and genetic properties of tumors associated with local immune cytolytic activity. *Cell* **160**, 48-61, doi:10.1016/j.cell.2014.12.033 (2015).

4 Barretina, J. *et al.* The Cancer Cell Line Encyclopedia enables predictive modelling of anticancer drug sensitivity. *Nature* **483**, 603-607, doi:10.1038/nature11003 (2012).
