# Supplementary material for: CRISPR activation screen identifies BCL-2 proteins and B3GNT2 as drivers of cancer resistance to T cell-mediated cytotoxicity
Source: Nat Commun. 2022 Mar 25;13:1606. doi: 10.1038/s41467-022-29205-8 (PMC8956604; doi:10.1038/s41467-022-29205-8)
Supplement: Supplementary file 2 — Reporting Summary [file 41467_2022_29205_MOESM2_ESM.pdf]

nature portfolio

Corresponding author(s): Julia Joung, Feng Zhang

Last updated by author(s): Feb 9, 2022

Reporting Summary

Nature Portfolio wishes to improve the reproducibility of the work that we publish. This form provides structure for consistency and transparency in reporting. For further information on Nature Portfolio policies, see our Editorial Policies and the Editorial Policy Checklist.

Statistics

For all statistical analyses, confirm that the following items are present in the figure legend, table legend, main text, or Methods section.

n/a	Confirmed	
☐	☒	The exact sample size (n) for each experimental group/condition, given as a discrete number and unit of measurement
☐	☒	A statement on whether measurements were taken from distinct samples or whether the same sample was measured repeatedly
☐	☒	The statistical test(s) used AND whether they are one- or two-sided<br>*Only common tests should be described solely by name; describe more complex techniques in the Methods section.*
☒	☐	A description of all covariates tested
☐	☒	A description of any assumptions or corrections, such as tests of normality and adjustment for multiple comparisons
☐	☒	A full description of the statistical parameters including central tendency (e.g. means) or other basic estimates (e.g. regression coefficient) AND variation (e.g. standard deviation) or associated estimates of uncertainty (e.g. confidence intervals)
☐	☒	For null hypothesis testing, the test statistic (e.g. F, t, r) with confidence intervals, effect sizes, degrees of freedom and P value noted<br>*Give P values as exact values whenever suitable.*
☒	☐	For Bayesian analysis, information on the choice of priors and Markov chain Monte Carlo settings
☒	☐	For hierarchical and complex designs, identification of the appropriate level for tests and full reporting of outcomes
☐	☒	Estimates of effect sizes (e.g. Cohen's d, Pearson's r), indicating how they were calculated

Our web collection on statistics for biologists contains articles on many of the points above.

Software and code

Policy information about availability of computer code

Data collection	TCGA2STAT 1.2
Data analysis	MAGeCK 0.5.7; g:Profiler (October 2, 2020); R 4.0.2; RSEM 1.3.1; GSEAPY 0.10.4; Bowtie 1.2.3; MACS 1.4.2; Python 2.7 and 3.7; FlowJo 10.8.1; Prism 8; Code for the analyses described in this study is available on Github (https://github.com/fengzhanglab/ Joung_Immunotherapy_Manuscript).

For manuscripts utilizing custom algorithms or software that are central to the research but not yet described in published literature, software must be made available to editors and reviewers. We strongly encourage code deposition in a community repository (e.g. GitHub). See the Nature Portfolio guidelines for submitting code & software for further information.

Data

Policy information about availability of data

All manuscripts must include a data availability statement. This statement should provide the following information, where applicable:

- Accession codes, unique identifiers, or web links for publicly available datasets
- A description of any restrictions on data availability
- For clinical datasets or third party data, please ensure that the statement adheres to our policy

The sequencing data generated in this study has been deposited in the Gene Expression Omnibus under accession code GSE159540. The mass spectrometry proteomics data has been deposited to the ProteomeXchange Consortium via the PRIDE partner repository with the accession code PXD031532. The Cancer Genome Atlas datasets were downloaded from the Broad GDAC Firehose (http://gdac.broadinstitute.org/) using the TCGA2STAT package for R. The human hg38 genome was downloaded from the UCSC Genome Browser (https://genome.ucsc.edu/). The remaining data are available within the Article, Supplementary Information, or Source Data file.

March 2021

Field-specific reporting

Please select the one below that is the best fit for your research. If you are not sure, read the appropriate sections before making your selection.

- [x] Life sciences
- [ ] Behavioural & social sciences
- [ ] Ecological, evolutionary & environmental sciences

For a reference copy of the document with all sections, see nature.com/documents/nr-reporting-summary-flat.pdf

Life sciences study design

All studies must disclose on these points even when the disclosure is negative.

Sample size	No statistical methods were used to predetermine sample size. For each assay, sample size was determined based on variability across independent experiments.
Data exclusions	No data were excluded.
Replication	Data represent experimental results that were reproduced at least once with either similar experimental setup or alternative assays.
Randomization	For animal studies, mice were randomized into test and control groups with or without adoptive cell transfer right after subcutaneous tumor injection. Randomization is not relevant to other cell culture-based experiments. The same number of cells were used for the experiments and the experiments were well-controlled.
Blinding	T cell injections were blinded between the test groups. While blinding to group allocation was not always possible due to COVID19 restrictions, the data collection and analysis were performed carefully by at least two different investigators.

Reporting for specific materials, systems and methods

We require information from authors about some types of materials, experimental systems and methods used in many studies. Here, indicate whether each material, system or method listed is relevant to your study. If you are not sure if a list item applies to your research, read the appropriate section before selecting a response.

Materials & experimental systems

n/a	Involved in the study	
	x	Antibodies
	x	Eukaryotic cell lines
x		Palaeontology and archaeology
	x	Animals and other organisms
	x	Human research participants
x		Clinical data
x		Dual use research of concern

Methods

n/a	Involved in the study	
	x	ChIP-seq
	x	Flow cytometry
x		MRI-based neuroimaging

Antibodies

Antibodies used	phospho- NF-kB p65 Ser536 (Cell Signaling Technology 3033S, 1:1,000), NF-kB p65 (Santa Cruz Biotechnology sc-8008, 1:200), phospho-STAT1 Tyr701 (Cell Signaling Technology 9167S, 1:1,000), STAT1 (Cell Signaling Technology 9172S, 1:1,000), CD276 (R&D Systems AF1027, 1:200), CD70 (Santa Cruz Biotechnology sc-365539, 1:200), CD58 (Thermo Fisher Scientific MA5800, 1:200), NECTIN2 (R&D Systems AF2229, 1:2,000), HLAA (Abcam ab52922, 1:5,000), TNFRSF1A (Santa Cruz Biotechnology sc-8436, 1:200), IFNGR2 (R&D Systems AF773, 1:200), FAS (Santa Cruz Biotechnology sc-8009, 1:200), IFNAR1 (Santa Cruz Biotechnology sc-7391, 1:100), TNFRSF10B (Novus Biologicals NB100-56618, 1:200), MICB (R&D Systems MAB1599-100, 1:500), TNFRSF10A (R&D Systems AF347, 1:200), PVR (R&D Systems MAB25301, 1:500), MICA (R&D Systems MAB1300-100, 1:500), HMGB1 (Abcam ab18256, 1:1,000), 4-1BBL (TNFSF9; R&D Systems AF2295, 1:200), NT5E (Abcam ab175396, 1:1,000), ULBP2 (R&D Systems AF1298, 1:2,000), IFNGR1 (R&D Systems MAB6731, 1:500), ULBP3 (R&D Systems AF1517, 1:2,000), CD39 (Abcam ab108248, 1:1,000), FLAG (Millipore Sigma F7425, 1:1,000), GAPDH (Cell Signaling Technology 2118L, 1:1,000), Anti-rabbit IgG-HRP (Cell Signaling Technology 7074S, 1:5,000), Anti-mouse IgG-HRP (Cell Signaling Technology 7076S, 1:5,000), anti-goat IgG-HRP (Santa Cruz Biotechnology sc-2354, 1:5,000), IgG Fc PE (Thermo Fisher Scientific 12-4998-82, 1:50), His Tag Alexa Fluor 647 (Thermo Fisher Scientific MA121315A647, 1:500), mouse Alexa Fluor 568 (Thermo Fisher Scientific A-11031, 1:400), or rabbit Alexa Fluor 647 (Thermo Fisher Scientific A-21244, 1:400)
Validation	All antibodies used in this study were validated by the manufacturers. Validation statements and literature citations are available on the manufacturer's websites.<br>phospho- NF-kB p65 Ser536 (https://www.cellsignal.com/products/primary-antibodies/phospho-nf-kb-p65-ser536-93h1-rabbit-mab/3033)<br>NF-kB p65 (https://www.scbt.com/p/nfkappab-p65-antibody-f-6)<br>phospho-STAT1 Tyr701 (https://www.cellsignal.com/products/primary-antibodies/phospho-stat1-tyr701-58d6-rabbit-mab/9167)<br>STAT1 (https://www.cellsignal.com/products/primary-antibodies/stat1-antibody/9172)

CD276 (https://www.rndsystems.com/products/human-b7-h3-antibody_af1027)
CD70 (https://www.scbt.com/p/cd27l-antibody-g-7)
CD58 (https://www.thermofisher.com/antibody/product/CD58-Antibody-clone-TS2-9-Monoclonal/MA5800)
NECTIN2 (https://www.rndsystems.com/products/human-nectin-2-cd112-antibody_af2229)
HLAA (https://www.abcam.com/hla-a-antibody-ep1395y-ab52922.html)
TNFRSF1A (https://www.scbt.com/p/tnf-r1-antibody-h-5)
IFNGR2 (https://www.rndsystems.com/products/human-ifn-gamma-r2-antibody_af773)
FAS (https://www.scbt.com/p/fas-antibody-b-10)
IFNAR1 (https://www.scbt.com/p/ifn-alpha-betaralpha-antibody-h-11)
TNFRSF10B (https://www.novusbio.com/products/trailr2-tnfrsf10b-antibody_nb100-56618)
MICB (https://www.rndsystems.com/products/human-micb-antibody-236511_mab1599)
TNFRSF10A (https://www.rndsystems.com/products/human-trailr1-tnfrsf10a-antibody_af347)
PVR (https://www.rndsystems.com/products/human-cd155-pvr-antibody-300907_mab25301)
MICA (https://www.rndsystems.com/products/human-mica-antibody-159227_mab1300)
HMGB1 (https://www.abcam.com/hmgb1-antibody-ab18256.html)
4-1BBL (TNFSF9; https://www.rndsystems.com/products/human-4-1bb-ligand-tnfsf9-antibody_af2295)
NT5E (https://www.abcam.com/cd73-antibody-ab175396.html)
ULBP2 (https://www.rndsystems.com/products/human-ulbp-2-5-6-antibody_af1298)
IFNGR1 (https://www.rndsystems.com/products/human-ifn-gamma-r1-cd119-antibody-92101_mab6731)
ULBP3 (https://www.rndsystems.com/products/human-ulbp-3-antibody_af1517)
CD39 (https://www.abcam.com/cd39-antibody-epr36782-ab108248.html)
FLAG (https://www.sigmaaldrich.com/US/en/product/sigma/f7425)
GAPDH (https://www.cellsignal.com/products/primary-antibodies/gapdh-14c10-rabbit-mab/2118)

Eukaryotic cell lines

Policy information about cell lines

Cell line source(s)	HEK293FT cells (Thermo Fisher Scientific R70007), A375 melanoma (Millipore Sigma 88113005-1VL), H1793 non-small cell lung adenocarcinoma (ATCC CRL-5896), H1299 non-small cell lung carcinoma (ATCC CRL-5803), LN-18 glioblastoma (ATCC CRL-2610), SK-N-AS neuroblastoma (ATCC CRL-2137), A2058 melanoma (ATCC CRL-11147), OAW28 ovarian cystadenocarcinoma (Millipore Sigma 85101601-1VL), SW1417 colorectal adenocarcinoma (ATCC CCL-238)
Authentication	Cell lines were obtained from manufacturers and not independently authenticated.
Mycoplasma contamination	Cell lines were confirmed negative for mycoplasma contamination by both manufacturer and authors.
Commonly misidentified lines (See ICLAC register)	None of the cell lines used are listed in the ICLAC register

Animals and other organisms

Policy information about studies involving animals; ARRIVE guidelines recommended for reporting animal research

Laboratory animals	Female NSG mice (strain 005557) aged 4-6 weeks were purchased from The Jackson Laboratory and used for tumor induction experiments.
Wild animals	study did not involve wild animals
Field-collected samples	study did not involve field-collected samples
Ethics oversight	The designs of animal studies and procedures were approved by the Institutional Animal Care and Use Committee (IACUC) of the Broad Institute.

Note that full information on the approval of the study protocol must also be provided in the manuscript.

Human research participants

Policy information about studies involving human research participants

Population characteristics	T cells were isolated from anonymous human healthy donors.
Recruitment	Leukapheresis products were purchased from the MGH blood bank.
Ethics oversight	Institutional Review Board protocol of the Massachusetts General Hospital

Note that full information on the approval of the study protocol must also be provided in the manuscript.

ChIP-seq

Data deposition

- [x] Confirm that both raw and final processed data have been deposited in a public database such as GEO.
- [x] Confirm that you have deposited or provided access to graph files (e.g. BED files) for the called peaks.

Data access links<br>*May remain private before publication.*	We have deposited the FASTQ and BED files to GEO (GSE159540).
Files in database submission	FASTQ and BED files for N-term and C-term FLAG JUNB, input and ChIP samples.
Genome browser session<br>(e.g. UCSC)	We can provide BAM and BED files for the genome browser session upon request.

Methodology

Replicates	Two biological replicates were used for each of the N-term and C-term FLAG JUNB. Only peaks that overlapped between the two FLAG versions were included for analysis
Sequencing depth	>60 million paired end reads were obtained for each sample. Samples had >85% alignment rate to the hg38 human genome.
Antibodies	anti-FLAG (Millipore Sigma F3165-1MG)
Peak calling parameters	MACS was run with command line options “-g hs -B -S --mfold 6,30”
Data quality	MACS was used to identify peaks and only peaks within 10kb of the transcriptional start site of genes were analyzed for overlap with RNA-seq.
Software	MACS and Python were used to analyze the data. Code will be provided upon request.

Flow Cytometry

Plots

Confirm that:

- [x] The axis labels state the marker and fluorochrome used (e.g. CD4-FITC).
- [x] The axis scales are clearly visible. Include numbers along axes only for bottom left plot of group (a 'group' is an analysis of identical markers).
- [x] All plots are contour plots with outliers or pseudocolor plots.
- [x] A numerical value for number of cells or percentage (with statistics) is provided.

Methodology

Sample preparation	Per condition, 500,000 cells were pelleted, washed with PBS, and fixed in 4% formaldehyde at 4C for 10 mins. Cells were washed twice with PBS and resuspended in PBS with the appropriate protein or antibody. Cells were incubated at 4C for 1h. Cells were washed with PBS and resuspended in PBS with the appropriate secondary antibody where applicable. Cells were incubated at 4C for 30 mins. Cells were washed twice with PBS before flow cytometry analysis. Please see the methods section for more details
Instrument	CytoFLEX Flow Cytometer (Beckman Coulter)
Software	FlowJo
Cell population abundance	Post-sort fractions were not analyzed.
Gating strategy	For each experiment, the same gating strategy for FSC/SSC was applied to all samples in a blinded manner. The median fluorescence intensities for each biological replicate are reported, with no additional gating.

- [x] Tick this box to confirm that a figure exemplifying the gating strategy is provided in the Supplementary Information.
