# Supplementary material for: CRISPR activation screen identifies BCL-2 proteins and B3GNT2 as drivers of cancer resistance to T cell-mediated cytotoxicity
Source: Nat Commun. 2022 Mar 25;13:1606. doi: 10.1038/s41467-022-29205-8 (PMC8956604; doi:10.1038/s41467-022-29205-8)
Supplement: Supplementary file 4 — Description of Additional Supplementary Files [file 41467_2022_29205_MOESM4_ESM.pdf]

Title: Supplementary Data 1
Description: MAGeCK analysis results of the CRISPRa screen for resistance to T cell cytotoxicity. Gene names, RefSeq IDs, and MAGeCK P-values for each of the screening bioreps are listed.

Title: Supplementary Data 2
Description: Negative control genes and candidate genes enriched in the CRISPRa screen. Set of 311 negative control housekeeping genes for estimating FDR of screening results and evaluating cytolytic activity. Negative control genes consisted of ribosomal proteins, RNA polymerases, translation factors, mitochondrial ribosomal proteins, GAPDH, and ACTB. Candidate genes were enriched in the top 1% across at least two screening replicates.

Title: Supplementary Data 3
Description: FDR of the CRISPRa screening results. Gene names, RefSeq IDs, and estimated FDR using negative control genes (see Methods) for each screening biorep are listed.

Title: Supplementary Data 4
Description: Pathway analysis of the 576 candidate genes. Pathways were ranked by FDR and pathways that overlapped (>30% of genes) with another pathway that had lower FDR were removed to identify distinct pathways.

Title: Supplementary Data 5
Description: MAGeCK analysis for genes that generally affect cell growth upon upregulation. Average MAGeCK P-values for cell growth or cell death analysis from 8 control screening replicates are listed. Candidate genes are annotated.

Title: Supplementary Data 6
Description: RNA-seq analysis of differentially expressed genes for candidate ORF overexpression.

Title: Supplementary Data 7
Description: ChIP-seq characterization of JUNB target genes. Only genes with JUNB ChIP-seq peaks that were significantly differentially expressed as measured by RNA-seq are listed.

Title: Supplementary Data 8
Description: Co-IP followed by mass spectrometry characterization of B3GNT2 target genes.
