# Supplementary material for: CRISPR activation screen identifies BCL-2 proteins and B3GNT2 as drivers of cancer resistance to T cell-mediated cytotoxicity
Source: Nat Commun. 2022 Mar 25;13:1606. doi: 10.1038/s41467-022-29205-8 (PMC8956604; doi:10.1038/s41467-022-29205-8)

Fig. 3f

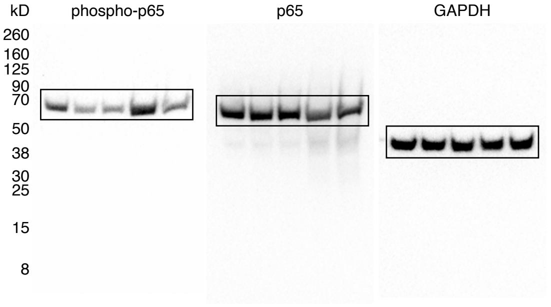

Fig. 4b

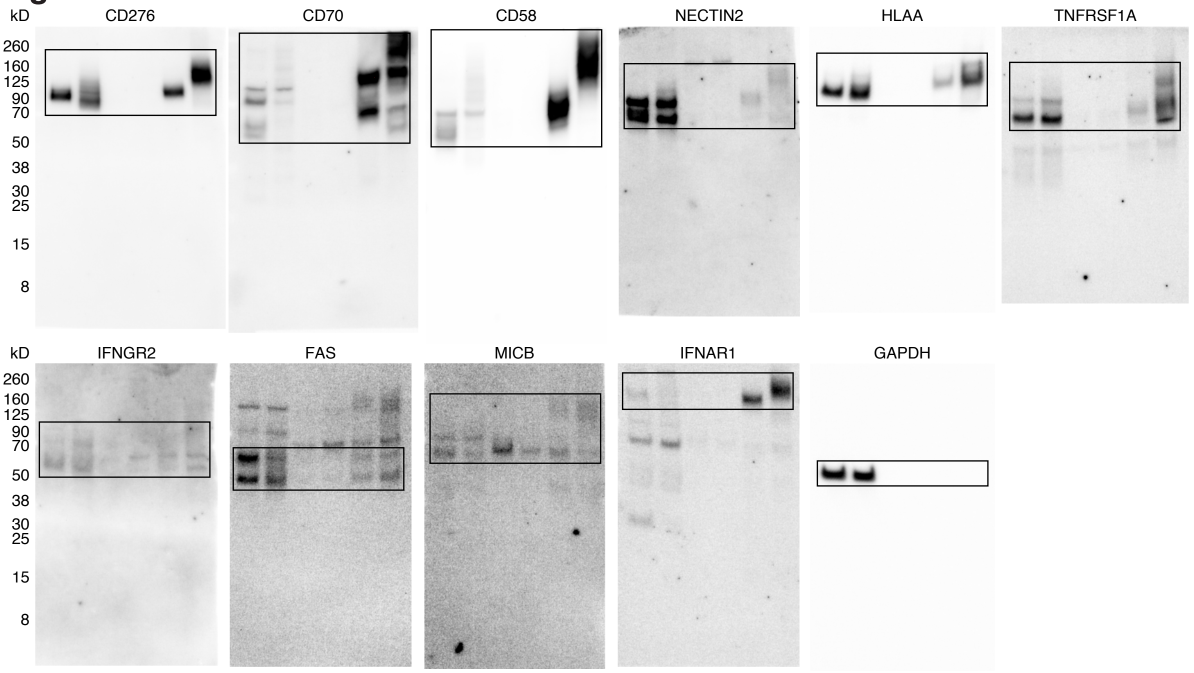

Fig. 4c

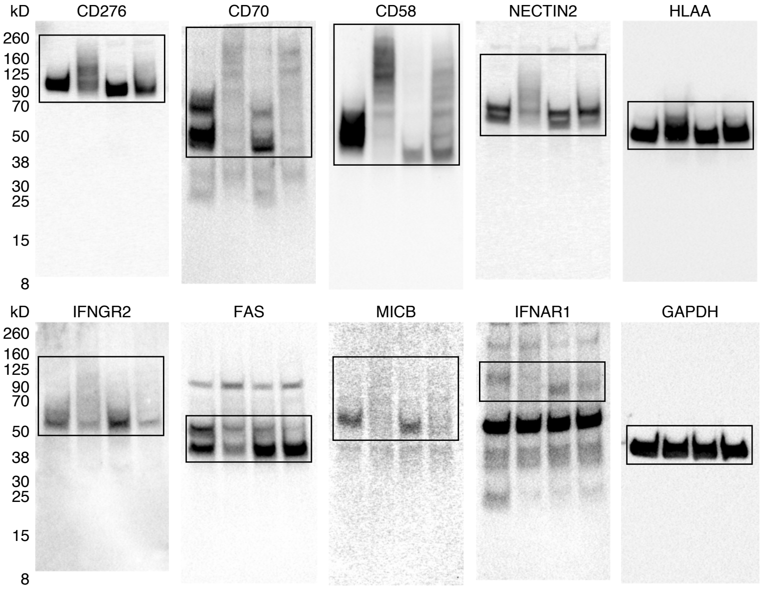

Supplementary Fig. 6d

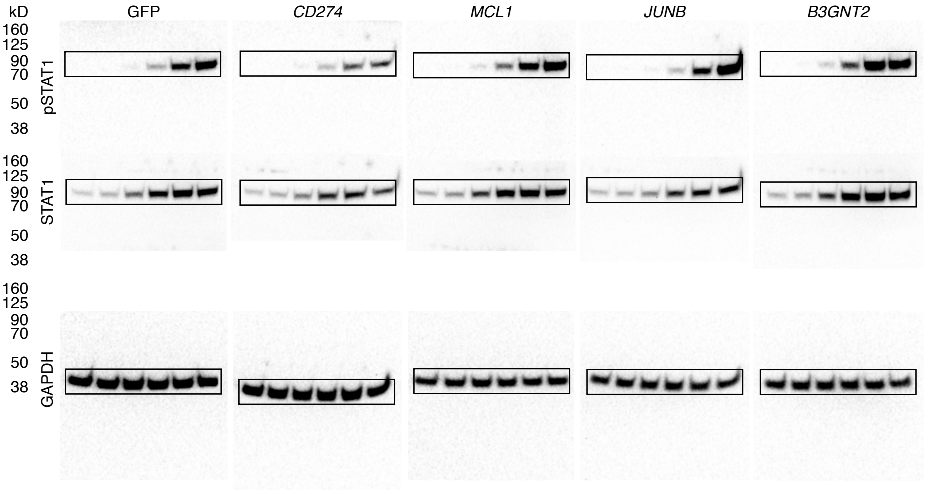

Supplementary Fig. 8c

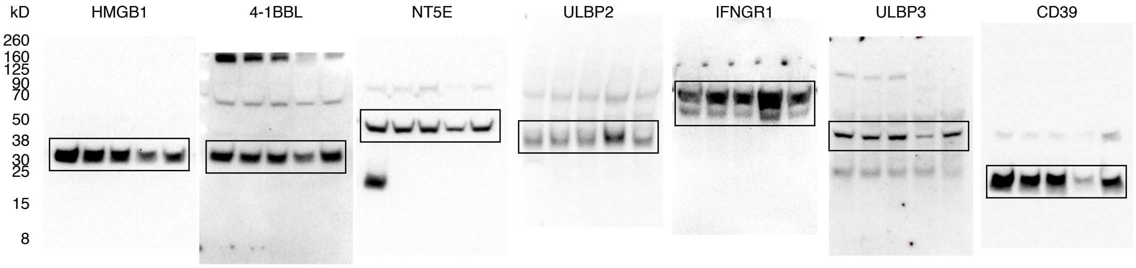

Supplementary Fig. 8d

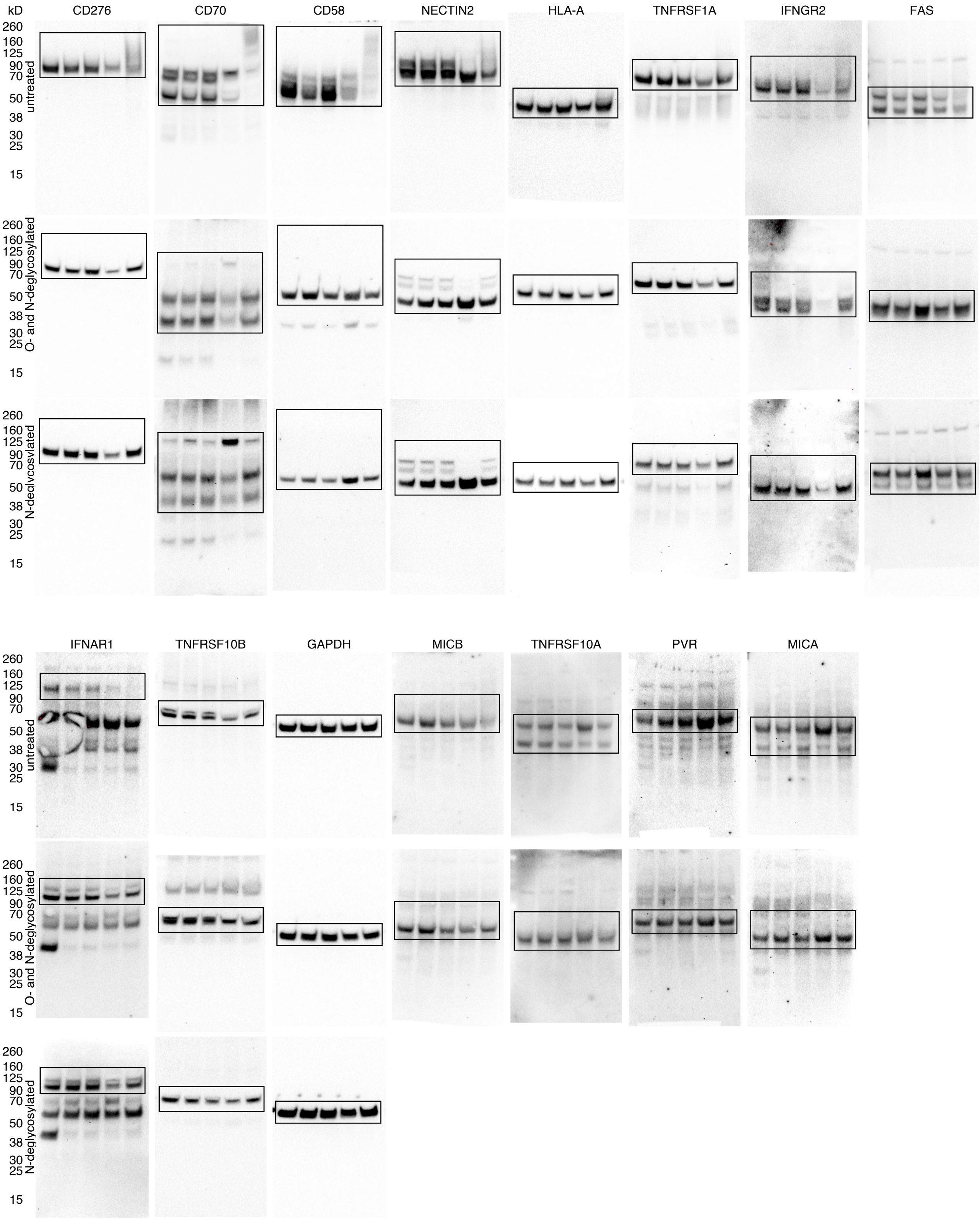

Supplementary Fig. 8e

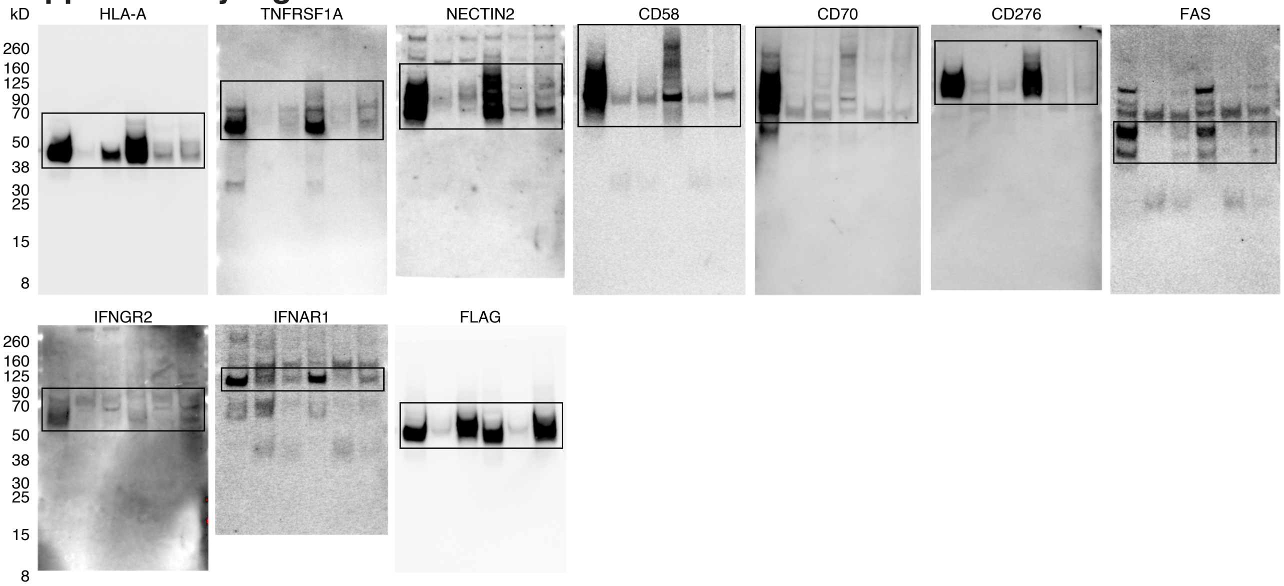

Supplementary Fig. 8f

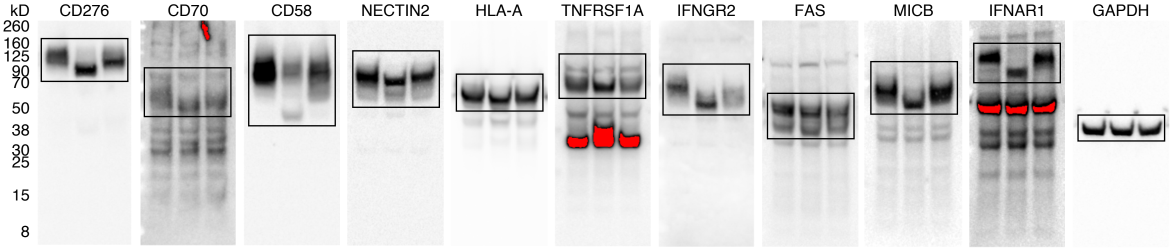

Supplementary Fig. 9d

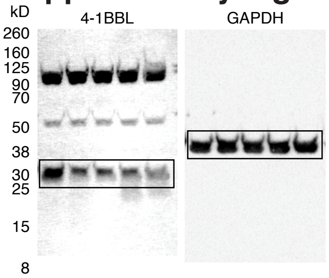

Supplementary Fig. 9g

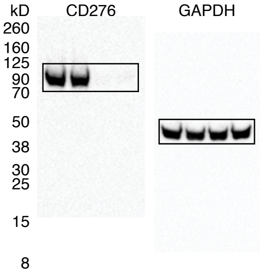

Supplement: Supplementary file 13 — Source Data [file 41467_2022_29205_MOESM13_ESM.zip › Source Data/uncropped blots.pdf]
